# A Multi-Source Sensor Dataset for Spain: Integrating Air Quality, Meteorological, Mobility and Calendar Records

**DOI:** 10.3390/s26123883

**Published:** 2026-06-18

**Authors:** Juan Bonastre-Egea, Andrés Bueno-Crespo, Juan Morales-García

**Affiliations:** 1 Escuela Politécnica Superior, Universidad Católica de Murcia (UCAM), 30107 Murcia, Spain; jbonastre@ucam.edu (J.B.-E.); abueno@ucam.edu (A.B.-C.); 2 Department of Software and Computing Systems, Universidad de Alicante (UA), 03690 Alicante, Spain

**Keywords:** open dataset, air quality monitoring, multi-source sensor integration, mobile network mobility data, meteorological sensors, environmental monitoring, nationwide, Spain

## Abstract

Air quality forecasting and environmental health research at urban and regional scales depend on the combination of measurements from heterogeneous sensor networks, yet the construction of integrated multi-source datasets is rarely described or released as a self-contained deliverable. This paper presents an open dataset that combines four sensor-derived sources covering the whole of Spain over the period from 2022 to 2024: hourly air quality observations from the 588 stations of the national network operated by the *Ministerio para la Transición Ecológica y el Reto Demográfico* (MITECO), daily meteorological records from the *Agencia Estatal de Meteorología* (AEMET), daily mobility indicators derived from anonymised mobile telephony events published by the *Ministerio de Transportes y Movilidad Sostenible* (MITMA) at the municipality level, and a calendar of national and Autonomous Community public holidays. The processing pipeline harmonises sources that differ in temporal resolution, spatial codification and quality regime into a tidy hourly table indexed by station and timestamp, with a fixed feature schema of 56 variables per record. Air quality stations are paired with their nearest AEMET station through a three-tier distance rule, and the daily exogenous features are aligned to the air quality time axis through a two-variant temporal-alignment scheme (lag-and-expand to the hourly grid for the hourly release, same-calendar-day join for the daily release). A complementary daily resolution variant of the dataset is also released, with 72 columns and the same feature schema except for the air quality block, which is aggregated to daily mean, minimum and maximum. The integrated dataset contains approximately 15 million hourly records across the 588 stations and is released on Zenodo (DOI 10.5281/zenodo.20196221) under a Creative Commons Attribution 4.0 International (CC BY 4.0) licence. It is intended as a substrate for research on air quality forecasting, environmental epidemiology and multi-source data fusion at the nationwide scale.

## 1. Introduction

Air pollution remains one of the most consequential environmental risks to public health in Europe: the European Environment Agency attributes hundreds of thousands of premature deaths each year to fine particulate matter, nitrogen dioxide and ground-level ozone, and the 2021 World Health Organization (WHO) air quality guidelines recently lowered the recommended thresholds for nearly every regulated pollutant [1,2]. Accurate short-term forecasts, as well as the analyses that underpin air quality policy, depend in turn on the quality of the data that feed them [3].

Our dataset integrates four sensor-derived sources that jointly characterise the atmosphere at each monitoring site: hourly concentrations of the regulated pollutants (NO_2_, NO_x_, O_3_, SO_2_, PM_10_, PM_2.5_, CO and benzene) from the 588 stations of the Spanish national network operated by the *Ministerio para la Transición Ecológica y el Reto Demográfico* (MITECO); daily meteorological summaries from the *Agencia Estatal de Meteorología* (AEMET), since temperature, humidity, wind and precipitation drive the emission and dispersion of pollutants [4,5,6]; municipality-level human mobility, since road traffic is among the largest proximate sources of urban pollution [7,8], derived by the *Ministerio de Transportes y Movilidad Sostenible* (MITMA) from anonymised mobile telephony records; and the national and regional public holiday calendar. Each source can be regarded as a sensor system in its own right, including the MITMA network, where mobile telephony events act as proxy observations of human movement.

The link between these sources and air quality is well established: commuting and freight traffic drive the diurnal and weekly cycles of NO_x_, CO and benzene, while weekends and public holidays depress these emissions and, by weakening NO titration, elevate ozone peaks (the weekend ozone effect) [7,8,9,10,11]. The COVID-19 lockdowns of 2020 made this link visible at scale, with NO_2_ dropping by 50% to 70% in major Spanish cities in step with vehicle mobility [12,13]; the 2022-to-2024 period released here reflects the normal post-lockdown operational regime.

These sources are heterogeneous in temporal resolution, spatial codification, missingness and quality regime, which makes their integration laborious. Most studies sidestep this by working with a single city and using either weather alone [5,6] or coarse mobility proxies such as traffic counters or closed-circuit television (CCTV) flows [9,14]; even richer city-level combinations [7,15,16] keep the integrated table inside a single pipeline rather than releasing it. As a result, reproducing or extending such work typically requires rebuilding the integration from scratch, often from incomplete descriptions of the temporal alignment and spatial association choices, which is both costly and a source of silent inconsistencies between studies. To our knowledge, no public release combines air quality, weather and mobile-network-derived mobility in a tidy, nationwide, station-level table spanning several years; releasing such a table, rather than the underlying observations alone, is the gap this paper addresses.

In order to address this gap, we release an integration pipeline and the resulting dataset for 2022 to 2024 (lower-bounded by the launch of the MITMA study), which performs distance-based pairing of air quality and meteorological stations, temporal alignment of the daily exogenous features, and a per-station enrichment join. Two variants are released side by side: an hourly variant (56 feature columns, ≈15 million records) at the native resolution of the air quality observations, and a daily variant (72 columns, ≈600,000 records) built with an unlagged join, intended for users whose downstream analysis already operates at daily resolution. The dataset is published on Zenodo under a Creative Commons Attribution 4.0 International (CC BY 4.0) licence [17], while the pipeline source code is released separately as an open repository on GitHub [18] so that the dataset can be regenerated, extended in time, or adapted to other countries with comparable open data. This release is aimed at researchers working on nationwide air quality forecasting, environmental epidemiology and the fusion of heterogeneous sensor data.

The remainder of this paper reviews related work (Section 2), describes the four sources and the integration pipeline (Section 3), specifies the released data records (Section 4), reports the technical validation (Section 5), offers usage guidance including preliminary modelling experiments (Section 6), and presents the conclusions (Section 7).

## 2. Related Work

This section reviews work relevant to the dataset described in this paper. We organise the discussion around three threads: (i) the landscape of open air quality data and the comparative absence of integrated multi-source releases, (ii) approaches that combine air quality with meteorological, mobility or other contextual sources, mostly at the urban scale, and (iii) the use of mobile-network-derived data as a sensor for human activity, both in general urban research and specifically in air quality studies.

### 2.1. Open Air Quality Data and the Integration Gap

Open air quality data have become considerably more accessible over the last decade, mostly through agency portals and through community platforms that harmonise heterogeneous national sources. The OpenAQ platform [19,20] aggregates real-time and historical observations from more than eighteen thousand monitoring locations across over a hundred countries, exposing a uniform API for measurements of the main regulated pollutants. At the European level, the e-Reporting infrastructure of the European Environment Agency [21] centralises the validated air quality data submitted by Member States and feeds the agency’s annual status assessments [2]. National administrations distribute their own raw observations in parallel, in formats that range from per-station REST APIs to bulk CSV downloads such as those of the Spanish MITECO network [22]. A complementary line of work explores how to densify these reference networks at urban scale through low-cost sensor platforms; Felici-Castell et al. [23], for instance, document an AI-enabled low-cost wireless sensor network deployed in the Valencian Community alongside the official regulatory stations, with deep neural network calibration of the raw readings and a 24 h forecasting horizon.

These resources share a common scope, however: they expose air quality measurements alone, or air quality plus locally co-located meteorology in the case of the urban sensor networks. Researchers wishing to combine them with mobility, land use or socio-demographic data must obtain each additional source from a different provider, harmonise temporal resolutions and spatial codifications by hand, and decide how to handle gaps and missingness across sources. The resulting integrated tables are typically internal to a single project’s pipeline and are not redistributed; reproducing a paper’s input data therefore requires reproducing the integration work, often from incomplete descriptions. A small number of dataset releases pair air quality measurements with weather or traffic counters at the city level, but to our knowledge, there is no public release that combines air quality, meteorology and mobile-network-derived mobility at the nationwide scale across multiple years.

This contribution is best understood in relation to the largest existing open air quality resources. Reference repositories such as the e-Reporting infrastructure of the European Environment Agency [21], the OpenAQ platform [19] and the U.S. EPA AirNow service expose pollutant measurements at continental or global scale [20], and the Copernicus Atmosphere Monitoring Service [24] additionally distributes modelled concentration and forecast fields; in every case, however, the released product covers a single domain, namely air quality observations or model output, at most accompanied by co-located meteorology. None of them ship the air quality signal pre-joined with mobile-network-derived mobility and a region-resolved public holiday calendar on a common station-level time axis. Unlike previous efforts that focused on single-source time series, our dataset combines high-resolution meteorological, mobility and holiday indicators with multi-station pollutant measurements across three full years and the entire national territory, so that the multi-source feature table that those repositories leave each researcher to assemble by hand is instead provided as a ready-to-use, reproducible deliverable. This integration, rather than the underlying observations themselves, is the novel and reusable element of the present release.

### 2.2. Multi-Source Data Fusion for Air Quality

A substantial body of work demonstrates that combining heterogeneous data sources can improve air quality estimation and forecasting, particularly in urban contexts. Early work by Yi et al. [15] introduced a distributed fusion network combining air quality stations, meteorology and points of interest at the city level in China. Subsequent contributions have extended this idea with attention-based encoders that align modalities at a fine temporal resolution [25] with transformer-style spatiotemporal architectures that operate over multi-source cyber-physical sensor streams [16], as well as with hybrid supervised and self-supervised models that adapt feature importance during training [26]. Recent multimodal models incorporate emission inventories alongside meteorology and pollutant observations [27], or fuse remote sensing imagery with ground-based measurements [28]. In all of these works, the input to the model is an integrated multi-source representation; in none of them is that integrated representation released as a self-contained dataset for reuse.

City-scale work in the same line illustrates how even modest source combinations improve forecasting accuracy and reveal interpretable patterns. Arsov et al. [29] report multi-horizon PM_10_ forecasts for Skopje using an LSTM-based recurrent architecture fed by historical air quality measurements from a network of city sensors and using daily meteorological summaries, showing systematic gains over an ARIMA baseline at the 6, 12 and 24 h horizons. Graça et al. [30] deploy a network of low-cost sensor stations together with co-located meteorological stations in the city of Aveiro and characterise the spatial and temporal patterns of NO_2_, PM and ozone across a full year, with traffic-hotspot stations exhibiting markedly different daily profiles from background sites. Cukjati et al. [31] combine ground-based IoT sensors with Sentinel-5P satellite retrievals through a Voronoi-cell ensemble of regression models, and produce gridded NO_2_ fields whose spatiotemporal resolution exceeds that of either input alone.

At the European and Spanish level, several studies illustrate the value of combining air quality with related sources at the urban scale. Folgado et al. [32] develop a deep learning model fed by air quality, meteorology and traffic counters for the city of Valencia. González-Enrique et al. [6] use exogenous meteorological variables to forecast NO_2_ in the Bay of Algeciras. Terroso-Sáenz et al. [33] extend their scope to the whole national network through a heterogeneous-graph neural network that exploits inter-station relations. Earlier hybrid deep learning frameworks combining pollution and weather inputs established the methodological baseline at the city scale [4,5]. The pattern across the literature is consistent: data fusion is well established as a useful direction, the model architectures continue to evolve, and the integrated datasets on which these models are trained remain largely undocumented and unreleased.

### 2.3. Mobile-Network Data as a Sensor for Human Activity

Mobile telephony has been studied for over a decade as a passive sensor for human movement. Calabrese et al. [34] provide an early and influential survey of urban sensing applications based on mobile phone network data, covering individual mobility, collective flows, presence estimation and event detection. The methodological backbone of this line of work is the extraction of origin–destination matrices from Call Detail Records (CDRs), where the relevant design choices (time-based versus routine-based aggregation, scaling to population totals, projection onto the road network) determine how faithfully the resulting flows reproduce reference traffic counts. Mamei et al. [35] compare prototypical implementations of the two main families of CDR-derived OD-matrix construction and evaluate them against ground-truth mobility surveys, providing a useful checklist of the assumptions that any downstream user of such matrices inherits. The Spanish national mobility study published by MITMA from 2022 onwards [36] belongs to this lineage: it is derived from anonymised events of the Orange Spain mobile network and reports daily aggregates of trips, overnight stays and people counts at the level of mobility zones that map to INE municipalities.

The use of mobility information as a covariate in air quality models is more recent, but several Spanish groups have already explored it. Morales-García et al. [7] introduce mobility-derived features into city-level pollution prediction models and report improvements over weather-only baselines, and subsequent work on the same line [37] examines how synthetic mobility data can supplement scarce real records. Hameed et al. [8] fuse air quality with traffic analytics in a multimodal deep learning framework, and Ibrahim and Lyons [9] show that even repurposed CCTV imagery can be converted into a city-scale NO_2_ signal. Xu et al. [14] propose interpretable multi-source pipelines that include mobile-derived flows alongside meteorology and emissions. Work on mobility forecasting itself with region-based flows and hierarchical spatial tessellation [38] is complementary to the use of mobility as an air quality predictor.

Multi-source sensor integration is also an active topic in adjacent environmental domains. Recent work on near-real-time integration of heterogeneous seismic feeds [39] illustrates how a similar pipeline philosophy can be applied to a different domain, with comparable challenges of temporal alignment and spatial association across independent agencies.

## 3. Materials and Methods

This section describes the four data sources that feed the integration pipeline and the procedure that combines them into the released dataset. Section 3.2 presents each source in turn, with summary statistics computed at their native resolution. Section 3.3 details the spatial association, temporal alignment and per-station enrichment operations that produce the final tidy table.

All data acquisition, processing and analysis were performed on an ASUS TUF Gaming F15 (model FX506LHB; ASUSTeK Computer Inc., Taipei, Taiwan) laptop. The integration pipeline was implemented in Python 3.12, using pandas (v3.0.1), numpy (v2.4.3), geopandas (v1.0.1), geodatasets (v2026.1.0), requests (v2.33.1), python-dateutil (v2.9.0), networkx (v3.6.1) and the holidays (v0.95) package. The figures presented in this paper were produced with matplotlib (v3.10.8) and seaborn (v0.13.2).

### 3.1. Overview of the Integration Pipeline

**Figure 1:**
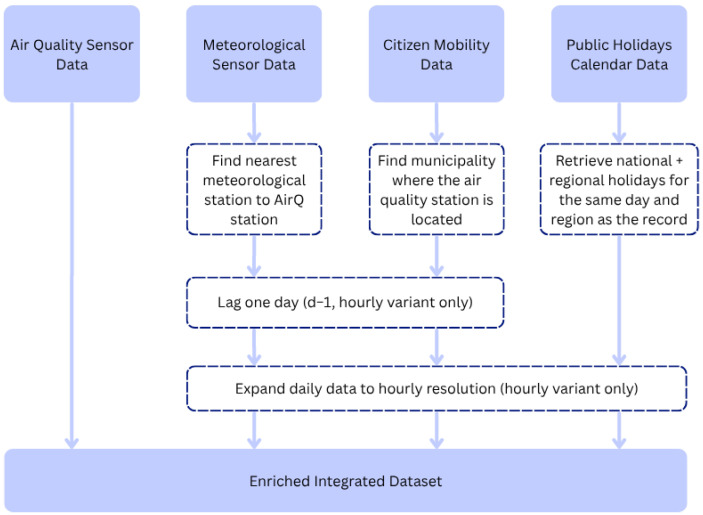
Overview of the multi-source data integration pipeline. The air quality series forms the spine of the dataset, and each exogenous source is spatially associated to the air quality station: the nearest AEMET station for meteorology, the containing municipality for mobility, and the station’s region and calendar day for the holiday calendar. In the hourly variant, the daily meteorological and mobility features are then lagged by one day (d−1) and expanded to hourly resolution before the join, whereas the holiday flags are assigned without lag. The result is the enriched integrated dataset. See Section 3.3 for a description of each stage.

The integration pipeline produces, for each station of the MITECO air quality network, a tidy table indexed by hourly timestamp and containing a fixed feature schema. Figure 1 summarises the pipeline, which is organised in the following four stages, depicting the spatial association of each exogenous source to the air quality station together with the one-day lag and daily-to-hourly expansion applied to the meteorological and mobility blocks in the hourly variant:

1. Metadata consolidation. The yearly station inventory files published by MITECO are read and deduplicated by station code, producing a single station registry that contains coordinates, province and municipality codes, the area type classification, and the dominant emission source label. The same operation is applied to the AEMET station inventory obtained from the OpenData API. The remaining two sources do not require a consolidation step at this stage: the MITMA mobility records are keyed directly by the INE 5-digit municipality code, which is constructed from the province and municipality codes already present in the MITECO registry, so no separate metadata file is needed and no acquisition of additional inventory data is performed; the public holiday calendar is keyed by date and Autonomous Community code, both of which are derived from the same MITECO registry through the official INE province-to-CCAA mapping.
2. Per-source acquisition and cleaning. Each of the four sources is acquired and processed independently through its own data interface. MITECO air quality data are downloaded as bulk annual CSV files from the agency’s open data portal; AEMET meteorological data are obtained from the OpenData REST API, with paginated calls respecting the maximum six-month window and the rate limit of approximately 50 requests per minute; MITMA mobility data are downloaded as monthly archives of compressed CSV files from the agency’s open mobility portal; and the public holiday calendar is constructed through the Python holidays package, which encodes both the national list and the Autonomous Community lists for the study period. The cleaning operations performed on each source are described in detail in Section 3.2.1, Section 3.2.2, Section 3.2.3 and Section 3.2.4. The output of this stage is a set of cleaned per-station and per-municipality time series with continuous datetime indices, one row per native time unit and one column per published variable.
3. Spatial association. Each MITECO air quality station is linked to the relevant record in every other source through identifiers derived from its inventory metadata. For meteorology, the MITECO and AEMET networks use independent station codes with no shared identifier, so each MITECO station is paired with the nearest AEMET station through a three-tier rule based on great-circle distance, computed by the Haversine formula [40]. The first tier accepts the nearest match if it lies within a 50 km threshold, the second tier restricts the search to the same INE province if the first tier fails, and the third tier accepts the nationwide nearest station as an unconditional fallback; the rule and the rationale for the 50 km cut-off are described in detail in Section 3.3.1, and the resulting empirical distribution of pair distances is reported in Section 5, where it is shown that every MITECO station in the network is matched under the first tier and the median pair distance is below 5 km. The output of this stage is a lookup table that records, for each MITECO station, the assigned AEMET station, the Haversine distance between the two and the tier under which the match was made. For mobility, the five-digit INE municipality code of each MITECO station is constructed by concatenating its province and municipality codes, and the MITMA records of that municipality are then attached to the station; all stations within the same municipality share the same mobility features. For holidays, the Autonomous Community code derived from the station’s province (through the official INE province-to-CCAA mapping) is used to retrieve the applicable regional holiday calendar in addition to the national one, which is the same for every station.
4. Per-station integration. For each MITECO air quality station, the pipeline assembles the integrated table by joining the air quality series with the meteorological, mobility and holiday records. The exact procedure differs slightly between the two temporal variants of the released dataset. In the hourly variant, the air quality observations remain at their native hourly resolution, and the daily meteorological and mobility records are first lagged by one day and then expanded to the 24 h of the corresponding day (the lag-and-expand procedure described in Section 3.3.2). In the daily variant, the air quality observations are previously aggregated to daily mean, minimum and maximum values, and the daily meteorological and mobility records are merged at the same calendar day without temporal shift. The holiday flags are assigned at time *t* without lag in both variants. The result is one file per station with a uniform schema across the network at either hourly or daily resolution.

Raw physical values are preserved throughout the pipeline. No normalisation, imputation or station filtering is applied at this stage, since these choices are properly left to users’ downstream analyses of the released dataset. The pipeline produces two temporal variants of the integrated dataset side by side. The hourly variant retains the native resolution of the air quality observations and uses the lag-and-expand alignment of the daily exogenous features described above. The daily variant first aggregates the hourly air quality series to daily mean, minimum and maximum statistics, and then joins the daily meteorological and mobility records at the same closed calendar day without any temporal shift, on the rationale that once a day has closed, its full weather and mobility summary is part of the same closed-day snapshot as the air quality aggregates. The aggregation requires at least 18 valid hourly readings out of 24 (75% coverage) for a daily value to be computed, following the data-capture criterion that Annex I of Directive 2008/50/EC [41] prescribes for valid daily averages of regulated atmospheric pollutants; days that fall below this threshold are marked as missing rather than aggregated from partial data. Both variants are released (Section 4).

### 3.2. Data Sources

The four data sources that feed the integration pipeline are described below. Each operates under a different institutional mandate, follows a different publication schedule, and uses a different spatial and temporal encoding; the operations that reconcile them are described in Section 3.3.

#### 3.2.1. Air Quality Data: The MITECO Sensor Network

The Spanish national air quality network is operated under the coordination of MITECO and brings together stations managed by the Autonomous Communities and several local administrations. The network reports hourly concentrations of the regulated pollutants (NO_2_, NO_*x*_, O_3_, SO_2_, CO, PM_10_, PM_2.5_, C_6_H_6_) following the European Air Quality Framework Directive, and publishes the data through annual CSV files with a uniform schema [22]. Each station is identified by a national code (COD_LOCAL) constructed from the INE province code, the INE municipality code and a station identifier. The metadata associated with each station include geographic coordinates (latitude, longitude, altitude), an area type classification (urban, suburban, rural) and a dominant emission source classification (traffic, industrial, background). Hours are reported in UTC and assigned at the end of the sampling interval.

For this work, we retrieved hourly data covering the years 2022 to 2024 for the 588 stations that were active for at least part of this period. The data are published as annual CSV files that can be downloaded from the MITECO web portal without API authentication. The native CSV format encodes one record per day, with one value column per hour (H01 to H24). The acquisition step pivots these wide records into a long hourly time series, joins the station metadata, and stores one file per station with a uniform schema. The processed dataset contains 14,944,488 station-hour records over the three-year window, an average of 25,416 valid records per station out of a theoretical maximum of 26,304 (96.6% temporal coverage on average). All eight pollutants are retained in the release, with substantially different coverage profiles across the network. NO_2_ and NO_*x*_ are the most widely deployed and are present at almost every station; O_3_, SO_2_ and PM_10_ are also broadly available; CO and PM_2.5_ are reported at roughly half the network; and C_6_H_6_ is the least common, present at a small subset of stations. Table 1 reports per-variable summary statistics for the eight pollutants.

The small negative minima for NO_2_, NO_*x*_ and PM_2.5_ correspond to sensor calibration drift near the detection limit, which is a normal artefact of in situ pollutant analysers. The values are retained in the release because the dataset preserves raw physical readings; a user wishing to remove them can do so trivially with a positivity filter.

**Figure 2:**
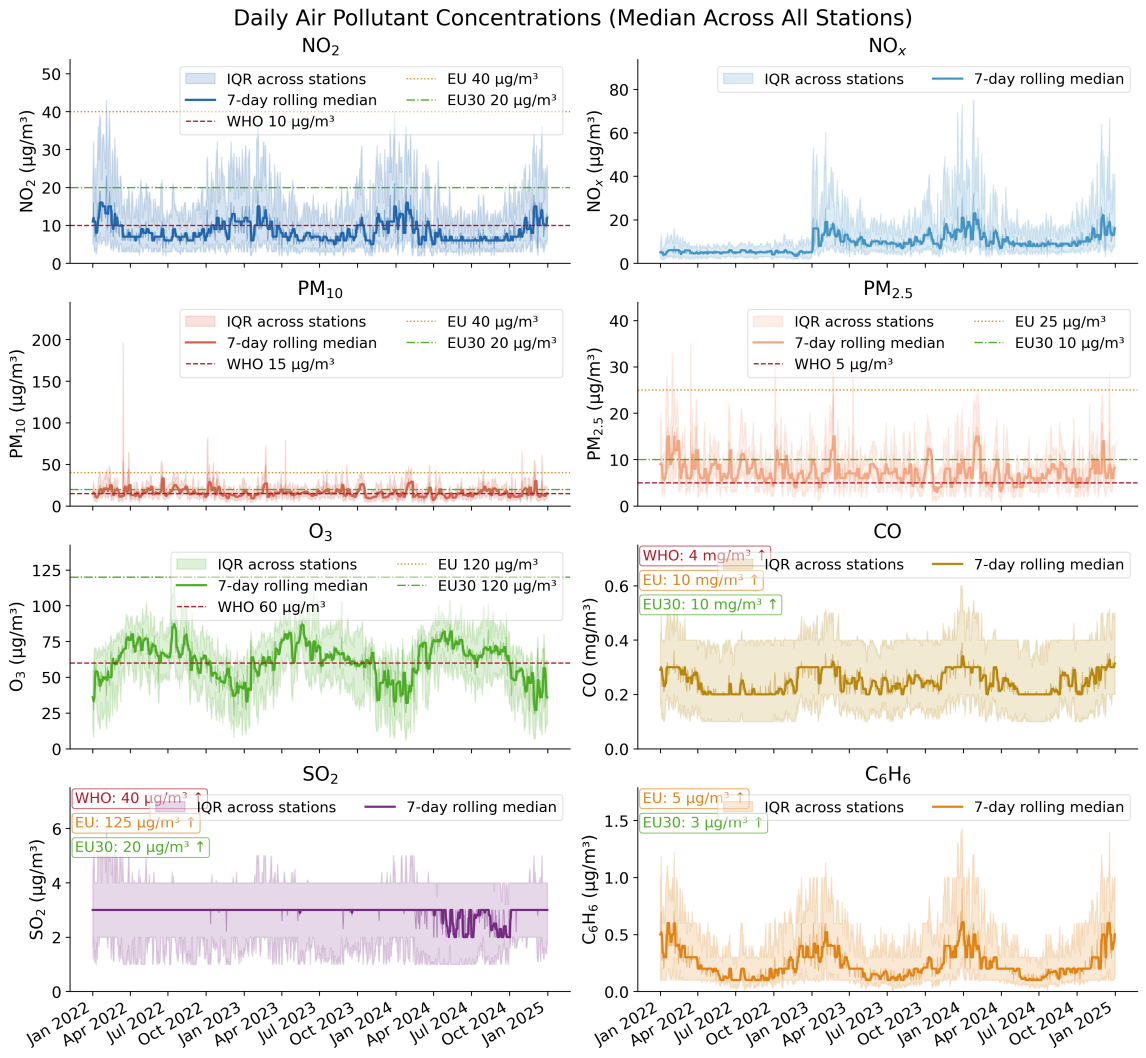
Daily median concentrations of the eight regulated air pollutants across the MITECO network over the 2022–2024 study period. For each pollutant, the solid line is the seven-day rolling median of the daily station-level medians, and the shaded band is the interquartile range across stations. Reference lines mark the WHO 2021 Air Quality Guideline (dashed), the current EU annual limit under Directive 2008/50/EC (dotted) and the revised EU 2030 limit under Directive (EU) 2024/2881 (dash-dot) where applicable. For pollutants whose regulatory limits are expressed on time bases other than the annual mean (CO, SO_2_, C_6_H_6_), the corresponding thresholds are annotated as text rather than as horizontal lines. When a reference value exceeds the upper limit of a panel’s vertical axis, its line falls outside the displayed concentration range and is therefore not drawn; this situation is indicated by an upward-pointing arrow next to the corresponding entry in the legend, so that the threshold remains documented even though it lies above the plotted range. Concentrations are in μg/m^3^ except for CO, which is in mg/m^3^.

Figure 2 shows the daily median concentrations of the eight pollutants of the MITECO block across the network. The seven-day rolling median is overlaid on the cross-station interquartile range, and reference lines are drawn for the WHO 2021 Air Quality Guideline, the current EU annual limit under Directive 2008/50/EC and the revised EU 2030 limit under Directive (EU) 2024/2881 where applicable.

The seasonal structure of the network differs markedly across pollutants. NO_2_ exhibits a clear seasonal cycle, with daily medians of 12 to 18 μg/m^3^ during autumn and winter (when stagnant atmospheric conditions, intensified domestic heating and sustained traffic emissions combine) and 5 to 10 μg/m^3^ during much of the warm season, hovering close to the WHO guideline of 10 μg/m^3^. The network-wide median stays well below the EU annual limit of 40 μg/m^3^ throughout the period, but the upper end of the cross-station distribution regularly approaches the new EU 2030 limit of 20 μg/m^3^ during cold-season episodes. NO_*x*_ follows a similar seasonal pattern at higher absolute concentrations.

PM_10_ has a flatter baseline near the WHO guideline of 15 μg/m^3^, with the network-wide median oscillating between 10 and 20 μg/m^3^. The series is punctuated by short, intense spikes that correspond to specific events: the most prominent peak in the record, in mid-March 2022, coincides with a major Saharan dust intrusion that affected the entire Iberian Peninsula and pushed the network-wide daily median above 50 μg/m^3^. The interquartile range across stations widens sharply during these episodes, indicating that not all areas are affected uniformly, and that PM_10_ exposure is governed as much by episodic regional transport phenomena as by chronic local emissions. PM_2.5_ follows a similar profile at lower absolute concentrations (typical median between 5 and 10 μg/m^3^), routinely exceeding the WHO guideline of 5 μg/m^3^ and frequently approaching the EU 2030 limit of 10 μg/m^3^.

Ozone shows the opposite seasonal pattern, with the highest concentrations in spring and summer (median between 70 and 90 μg/m^3^), driven by photochemical production under intense solar radiation, and the lowest in winter (40 to 50 μg/m^3^). The WHO peak-season threshold of 60 μg/m^3^ is exceeded by the network-wide median throughout the warm half of each year, although the EU and EU 2030 limit of 120 μg/m^3^ is rarely reached at the network-wide level. CO, SO_2_ and benzene remain comfortably below their respective regulatory thresholds across the entire period: CO oscillates around 0.2 to 0.4 mg/m^3^ against a WHO 24 h guideline of 4 mg/m^3^; SO_2_ stays close to 2 to 3 μg/m^3^, two orders of magnitude below the EU daily limit; and benzene varies between 0.1 and 0.6 μg/m^3^ against an EU annual limit of 5 μg/m^3^, with a clear seasonal modulation that elevates winter concentrations in line with NO_2_ and CO.

**Figure 3:**
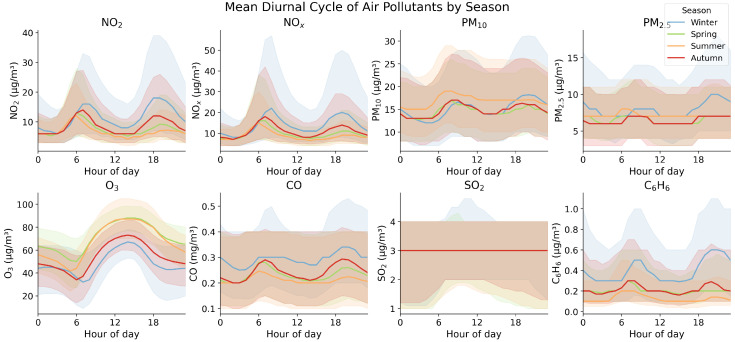
Mean diurnal cycle of the eight regulated pollutants by season, computed across the network for the years 2022–2024. Solid lines are seasonal means of the hourly station-level means; shaded bands span the 25th to 75th percentile of the cross-station distribution. CO is in mg/m^3^; all other pollutants are in μg/m^3^.

Aggregating the hourly observations by season exposes the diurnal cycle of each pollutant (Figure 3). NO_2_ and NO_*x*_ both show a clear bimodal pattern with a morning peak around 07:00–08:00 and a stronger evening peak between 19:00 and 21:00, both compatible with the temporal structure of the urban traffic load and amplified in winter by the lower mixing-layer height and reduced photolytic loss. The winter cycle of NO_2_ spans from a minimum around 10 μg/m^3^ in the early hours to an evening peak of approximately 26 μg/m^3^, while in summer, the same cycle contracts to roughly 7–16 μg/m^3^ as boundary-layer mixing and photochemical sinks weaken its diurnal accumulation. CO and benzene mirror the NO_2_ profile, with rush-hour peaks that are most clearly defined in winter, in line with their shared origin in combustion processes.

Ozone exhibits the inverse pattern, with a single broad afternoon maximum centred around 14:00 in every season, reaching about 87 μg/m^3^ in spring and summer when photochemical production is at its highest, and a pronounced morning minimum coinciding with the NO_2_ peak. The morning minimum reflects both the titration of ambient ozone by freshly emitted NO and the dominance of dry deposition during the nocturnal stable layer.

The particulate pollutants behave seasonally rather than uniformly. PM_10_ has its most pronounced diurnal cycle in winter, with a bimodal pattern that mirrors the combustion-driven shape of NO_2_: a morning shoulder around 09:00 near 24 μg/m^3^ and a stronger evening peak close to 20:00 reaching 27 μg/m^3^ against an early-hours minimum of about 18 μg/m^3^. In the other three seasons, the cycle is markedly flatter, with a single morning peak around 07:00–08:00 and an overall amplitude roughly half of that observed in winter. PM_2.5_ follows the same seasonal contrast in a more extreme form: in winter, it traces a clear bimodal cycle with an evening peak of approximately 14 μg/m^3^ against a minimum near 9.5 μg/m^3^, whereas in spring, summer and autumn, it remains nearly flat throughout the day, with intra-day variation within 1–2 μg/m^3^. SO_2_ shows no significant intra-day structure in any season, consistent with its dominant industrial point sources and with the fact that most stations report values close to the detection limit. For users of the dataset, the practical implication is that the within-day variation in every pollutant other than SO_2_ is non-negligible in at least one season, whereas the daily aggregated exogenous features described in the following subsections cannot describe within-day variation by construction.

**Figure 4:**
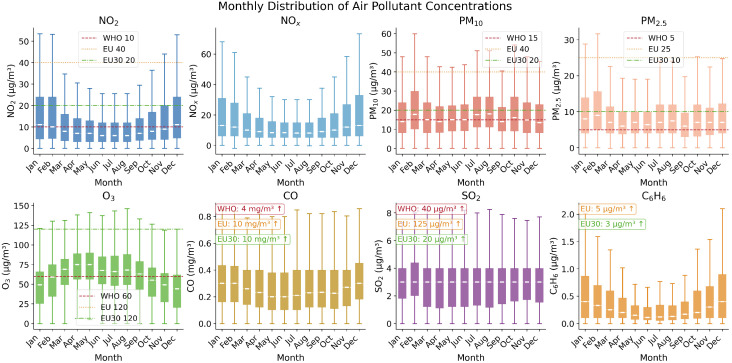
Monthly distribution of the eight regulated pollutants pooled across all station-hour records of the network over 2022–2024. Each box spans the interquartile range with the median marked; whiskers extend to the 1.5 IQR limits. Reference lines mark the WHO 2021 Air Quality Guideline (dashed), the current EU annual limit (dotted) and the revised EU 2030 limit (dash-dot) where they fall within the panel range. When a threshold exceeds the upper limit of a panel’s vertical axis, it is not drawn as a horizontal line but is instead annotated at the upper margin in a bordered box, with an upward-pointing arrow indicating that the value lies above the displayed range. For O_3_, the current EU limit and the revised EU 2030 limit share the same threshold (120 μg/m^3^) and therefore coincide as a single dash-dot reference line; no separate orange dotted line appears in that panel. CO is in mg/m^3^; all other pollutants are in μg/m^3^.

The same seasonal structure can be summarised compactly through the monthly distribution of each pollutant (Figure 4). NO_2_ and NO_*x*_ peak in December and January, with monthly upper quartiles reaching 24 μg/m^3^ and 33 μg/m^3^, respectively, and a trough in June through August at roughly half of those values. Ozone shows the opposite cycle, with the highest medians in April and May (close to 75 μg/m^3^) and a slightly lower summer plateau of 66 to 68 μg/m^3^ in June through August, declining sharply through autumn into a winter minimum; the upper whiskers occasionally approach the EU and EU 2030 threshold of 120 μg/m^3^ during the warm season. Benzene reproduces the NO_2_ winter peak at much lower absolute concentrations (medians of 0.40 μg/m^3^ in December and January against 0.11 μg/m^3^ in June), consistent with their shared combustion origin. PM_10_ and PM_2.5_ are comparatively flat across the calendar, with February being the most variable month for both, as judged by the interquartile spread, reflecting the combination of winter combustion peaks and the onset of Saharan dust transport. CO follows the NO_2_ winter peak weakly. SO_2_ shows no monthly structure, in line with its largely industrial sources.

#### 3.2.2. Meteorological Data: The AEMET Sensor Network

AEMET operates the official meteorological network of Spain and exposes its daily climatological summaries through the OpenData API [42]. The relevant product for this work is the daily climatological extract per station (*climatologias diarias*), which reports temperature (mean, minimum and maximum), precipitation, wind speed (mean and gust), wind direction, insolation, atmospheric pressure (maximum and minimum) and relative humidity (mean, minimum and maximum). The API enforces a maximum window of six months per request and a rate limit of approximately 50 requests per minute, both of which the acquisition module respects through paginated calls and throttling. The processed dataset contains 990,784 station-day records from 904 AEMET stations over the 1096-day window after deduplication of repeated identifiers. From this catalogue, we retain the nine variables that constitute the meteorological block of the integrated dataset: mean, minimum and maximum daily temperature (temp_mean, temp_min, temp_max); daily precipitation (precip); mean and maximum wind speed (wind_speed, wind_gust); and mean, minimum and maximum relative humidity (humidity_mean, humidity_min, humidity_max). Wind direction, solar radiation, atmospheric pressure and timing metadata are dropped at the download stage. The retained variables are uniformly well covered across the catalogue, with missingness of approximately 5% for all nine and a profile of mean values consistent with the climatic range of Spain. Table 2 reports per-variable summary statistics. The Spanish convention “Ip” (*indicios de precipitación*, trace amounts of rainfall) is converted to 0.0 at the acquisition stage. The integration of these daily records onto the hourly air quality time axis used by the merged release follows the lag-and-expand procedure described in Section 3.3.2: each daily summary is shifted forward by one day and replicated across the 24 h of the corresponding day in the hourly variant; the daily variant of the merged dataset uses the same daily values without temporal shift.

The 111% maximum for humidity_max and the −50 °C minimum for temp_max are present in the source records as published by AEMET, and the dataset preserves them as released.

#### 3.2.3. Mobility Data: The MITMA Sensor-Derived Network

The MITMA mobility study is a continuously updated nationwide mobility dataset for Spain derived from mobile telephony records [36]. The study processes anonymised event records from approximately 13 million Orange Spain mobile lines through a hashing-based pseudonymisation function and aggregates the resulting trajectories into mobility indicators with daily temporal resolution. MITMA publishes the results at multiple zonifications; we use the municipality-level aggregation, which covers 2687 zones, each constructed from one or several INE census districts. The methodology combines passive network probe events with Call Detail Records, producing trip matrices that include an intra-day hourly breakdown, but the published aggregate files are daily studies that characterise mobility patterns for each calendar day as a whole.

Three indicator families per zone and per day are released by MITMA and incorporated into the integrated dataset. The *pernoctaciones* product counts the number of people who spent the night in a zone, distinguishing residents from visitors. The *personas* product counts people present in a zone segmented by age band and by number of trips made that day. The *viajes* product counts trips with their origin or destination in the zone, segmented by trip purpose (home, work or study, frequent activity, infrequent activity), by household income level, by age band and by residency status. These products are published as monthly archives of daily files in a compressed CSV format and were downloaded from the MITMA open data portal. After acquisition and pivoting, the processed mobility dataset contains 2,824,332 zone-day records for the *viajes* block and 2,781,949 zone-day records for each of the *personas* and *pernoctaciones* blocks over the 1096 calendar days of the study period, with no missing values at the zone-day level. The raw trip matrices include an intra-day hourly breakdown (a period field with 24 *franja* values), but counts are aggregated to the day level so that the resulting indicators describe the daily context of each station rather than within-day traffic dynamics. Table 3 reports per-variable summary statistics for the three blocks. Mean values across the network are dominated by the largest urban zones; medians give a better sense of the typical zone-day, as the distributions are strongly right-skewed.

The integration step assigns the mobility indicators of the INE 5-digit municipality code corresponding to each station, constructed by concatenating the province and municipality codes from the station inventory. An official cross-reference file (*relacion_ine_zonificacion_mitma*) published by MITMA is used to translate from the internal MITMA zone identifiers to INE codes. As a consequence, all air quality stations located within the same municipality share identical mobility features. This is a known approximation: intra-municipal variability of mobility patterns is not captured, although the spatial heterogeneity of large urban municipalities should be small relative to the inter-municipal differences that the indicators are designed to express. Each daily mobility record is then aligned to the air quality time axis following the same convention used for the meteorological features: in the hourly variant, the record is lagged by one day and expanded to the 24 h of the corresponding day, while in the daily variant, it is merged directly on the same calendar day without temporal shift. The two alignments are described in Section 3.3.2.

**Figure 5:**
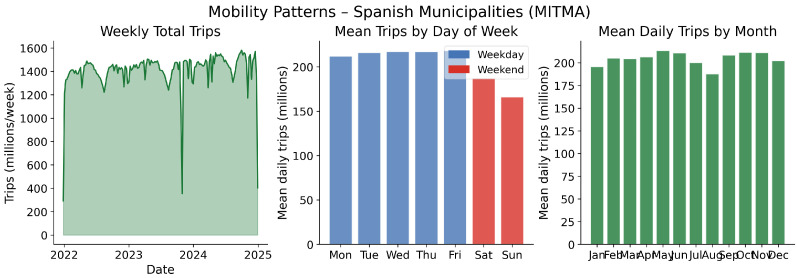
Aggregate mobility patterns over the 2022–2024 period, summed across all 2687 zones of the municipal-level MITMA zonification. Left: Total weekly trips. Centre: Mean daily trip count by day of the week, with weekdays (blue) and weekends (red) distinguished. Right: Mean daily trip count by month of the year.

Figure 5 summarises the temporal structure of the mobility data at the national level. The aggregate weekly trip volume is remarkably stable at around 1.4 to 1.5 billion trips per week throughout the three years of the study, with sharp drops to fewer than 600 million during the two weeks of late December that contain Christmas Day and New Year (covering the transitions from 2021 to 2022 and from 2024 to 2025) and over a single week at the end of October 2023, whose unusually low aggregate sits on the boundary of two MITMA monthly batches and most likely reflects a partial-data effect rather than a real mobility collapse. The day-of-week breakdown reveals a steady weekday baseline of approximately 215 million daily trips and a noticeable contraction on weekends (around 187 million on Saturdays and 166 million on Sundays, the latter representing a roughly 23% reduction relative to the weekday mean). The monthly profile is comparatively flat, with a modest dip in August reflecting the Spanish summer-vacation convention.

#### 3.2.4. Public Holiday Calendar

National and regional public holidays are obtained from the open-source Python package holidays [43], which compiles official holiday calendars per country and per subnational region. For Spain, the package covers national holidays and the calendars of all 17 Autonomous Communities plus the autonomous cities of Ceuta and Melilla, identified by their two-letter ISO codes (for example, AN for Andalusia, MD for Madrid, CT for Catalonia, PV for the Basque Country, and MC for the Region of Murcia); the finest spatial granularity available is at the Autonomous Community level, not the province. Across the three-year window, we record 26 unique national holiday dates (8 in 2022, and 9, respectively, in 2023 and 2024) and 83 unique regional dates that, when expanded across the Autonomous Communities to which they apply, yield 204 region-specific holiday entries. Most communities contribute around ten distinct regional dates over the period.

The integration step generates two binary indicators per station and per timestamp: national_holiday, which equals one if the date is a national public holiday, and regional_holiday, which equals one if the date is a holiday specific to the Autonomous Community in which the station is located. Both indicators are retained in the release. Although their content overlaps in part (every national holiday is also a holiday in every Autonomous Community), the regional indicator captures dates that the national one does not, and the marginal cost of carrying it as an additional binary column is negligible. In contrast to the meteorological and mobility features, the holiday indicators are assigned at time *t* without any temporal shift, since the calendar of public holidays is known in advance.

**Figure 6:**
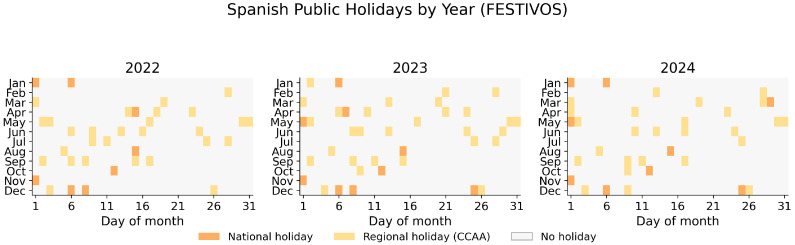
Calendar of Spanish public holidays for 2022, 2023 and 2024. Orange cells mark national holidays; yellow cells mark days that are public holidays in at least one Autonomous Community.

Figure 6 displays the calendar of national and regional holidays for the three years of the study period. National holidays (orange) cluster on a recurring set of civil and religious dates (New Year, Epiphany, Good Friday, Labour Day, the Assumption, Spain’s National Day, All Saints, Constitution Day, the Immaculate Conception and Christmas), while the regional component (yellow) is denser and irregularly distributed across the calendar, with each Autonomous Community contributing a small set of dates that reflect local historical or religious observances. This figure confirms that the regional indicator is not redundant with the national one for a large share of the year, particularly during late winter, spring and early autumn.

#### 3.2.5. Source-Level Summary

The four sources described above produce datasets with substantially different shapes and granularities. Table 4 summarises the high-level properties of each, as observed at its native resolution after acquisition and per-source cleaning, before the integration step that brings everything onto the common hourly station grid described in Section 3.3.

The asymmetry in spatial and temporal granularity is the central characteristic that the integration pipeline must reconcile: hourly station data on one side, daily zone or station data on the other, and a calendar of dates on the third. Section 3.3 describes how the pipeline brings these levels into a single tidy table.

### 3.3. Integration Procedure

The integration procedure takes the per-source cleaned tables of Section 3.2 and produces one CSV file per air quality station, indexed by hourly timestamp and containing the full feature schema. Three operations comprise the procedure: the spatial association of each MITECO station with the other sources (meteorology by distance-based pairing, mobility and holidays by administrative code lookup), the temporal alignment of the daily exogenous features to the hourly grid, and a per-station enrichment join that brings all the blocks together.

#### 3.3.1. Distance-Based Pairing of Air Quality and Meteorological Stations

Among the three spatial associations performed by the pipeline, the link between the MITECO and AEMET stations is the only one that is not reduced to a lookup by administrative code: the mobility and holiday linkages use the INE municipality code and the province-to-CCAA mapping, respectively, and are described together with the corresponding sources in Section 3.2.3 and Section 3.2.4. The meteorological linkage, in contrast, must reconcile two independent networks that share no identifier. Each MITECO station is paired with exactly one AEMET station rather than with a weighted average of several neighbours; this one-to-one assignment keeps the feature schema fixed across the network and avoids the sparse missing-value patterns that an averaging scheme would introduce when different MITECO stations sample different sets of AEMET sources. Both station lists are pre-filtered to include only stations with a data file on disk, preventing assignments to AEMET stations with no usable record.

**Figure 7:**
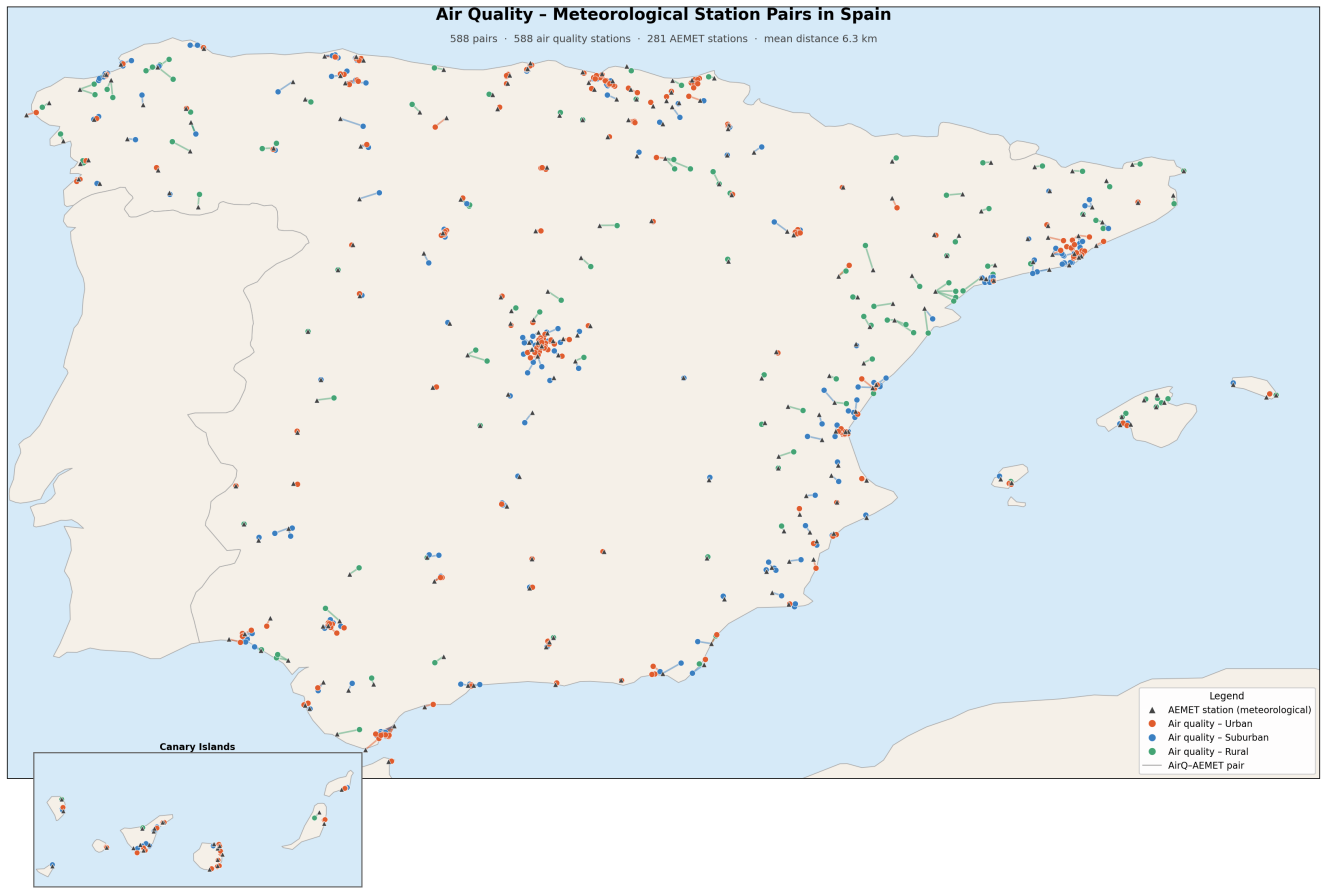
Geographic distribution of the 588 MITECO air quality stations, classified by area type (urban, suburban, rural). The lines connect each air quality station to the AEMET meteorological station assigned by the three-tier spatial association procedure described in Section 3.3.1.

The pairing follows a three-tier rule based on Haversine distance. For each MITECO station, the closest AEMET station is identified; if it lies within a 50 km threshold, the match is accepted (priority 1, label *distancia*). If no AEMET station lies within 50 km, the search is restricted to stations in the same province (matched by accent-stripped name) and the closest one is selected (priority 2, label *provincia*). If no AEMET station exists in the same province, the closest one nationwide is assigned regardless of distance (priority 3, label *fallback_absoluto*). The 50 km threshold is a compromise between proximity and coverage: lower thresholds leave stations in sparsely instrumented interior provinces unmatched, while higher thresholds allow pairs that we judged too distant to share a similar mesoscale meteorological state. In practice, the AEMET network is dense enough that every MITECO station in the release is matched under the first tier; the empirical distribution of pair distances is reported in Section 5 and has a median of 4.8 km, a mean of 6.3 km and a maximum of 30.1 km, so the two fallback tiers are not exercised in the present release although they remain part of the pipeline for robustness against future MITECO additions in sparsely instrumented areas. The result is published alongside the dataset as a lookup table that records, for each MITECO station, the assigned AEMET station, the Haversine distance between the two, and the tier label of the assignment. Figure 7 shows the geographic distribution of the matched pairs.

#### 3.3.2. Temporal Alignment of Daily Exogenous Features

Air quality is reported hourly while meteorology, mobility and holidays are reported daily. The integration brings the three daily sources onto the air quality time axis through a procedure that depends on the temporal resolution of the released variant.

In the hourly variant, the daily meteorological and mobility records are first shifted forward by one day (lag d−1) and then expanded to the 24 h of the corresponding day by repetition. This lag reflects the operational forecasting constraint where, at the moment of issuing a forecast for time *t*, the most recent complete daily summary of weather and mobility available is the one that closed at the end of the previous day; this shift therefore prevents future information from leaking into the hourly record at time *t*. The combination of lag and expansion preserves the operational validity of every record and produces a uniform hourly schema, at the cost of the redundant repetition of each daily value across the 24 h of its day.

In the daily variant, the air quality series is first aggregated to the daily grid (per-pollutant daily mean, minimum and maximum, with the 75% data-capture rule introduced in Section 3.3), and the daily meteorological and mobility records are then merged at the same calendar day as the air quality aggregates without temporal shift. The operational rationale for the lag does not apply at this resolution: once a calendar day has closed, its complete weather and mobility summary is part of the same closed-day snapshot as the air quality aggregates, so applying a d−1 shift would discard one day of valid information without operational justification. The daily variant therefore aligns every feature block on the same closed-day timestamp.

Holiday flags are assigned at time *t* without lag in both variants, since the public holiday calendar is known in advance and is therefore available to any forecaster issuing a prediction at any time during the day.

#### 3.3.3. Per-Station Enrichment Join and Feature Schema

For each MITECO station, the pipeline assembles the integrated table as follows. The hourly air quality series is loaded and reindexed to a continuous hourly DatetimeIndex over the study period, with gaps filled with NaN to preserve the time axis. The AEMET counterpart of the station, looked up in the spatial pairing table, supplies the daily meteorological record, which is shifted by d−1 and expanded to hourly resolution, as described above. The municipality-level MITMA mobility record (pernoctaciones, personas and viajes blocks) supplies the daily mobility indicators, which undergo the same lag-and-expand operation; if no MITMA file exists for the municipality (a few very small municipalities are not covered by MITMA), the corresponding columns are filled with NaN. The holiday flags are computed for each timestamp from the calendar of national and Autonomous Community holidays using the province-to-CCAA mapping derived from the station inventory. Finally, the station’s area type label (area_type) is appended as a constant categorical column. The first calendar day of the series is discarded from the output because the d−1 lag of the mobility and meteorological features would require a record from the day before the series starts, which is NaN by construction.

The resulting per-station table contains 56 feature columns indexed by hourly timestamp, grouped as summarised in Table 5. The same schema is produced for every station, regardless of which subset of pollutants the station reports; pollutants not measured at a given station appear as columns of NaN, which keeps the schema uniform across files and allows downstream consumers to scan stations by column without conditional logic.

The resulting dataset comprises approximately 15 million hourly records distributed across the 588 stations of the network, released as a set of per-station CSV files. The full released artefact, including all per-station files, the spatial pairing lookup table and the source-level metadata files, is described in Section 4.

## 4. Data Records

The dataset described in this paper is released as an open record on the Zenodo repository under a Creative Commons Attribution 4.0 International (CC BY 4.0) licence [17]. The persistent identifier of the record is 10.5281/zenodo.20196221, and the record can be reached at https://doi.org/10.5281/zenodo.20196221. Future revisions of the dataset are deposited as new versions of the same Zenodo record; the cited concept DOI always resolves to the latest published version. The deposit contains all per-station files of the integrated dataset, the corresponding cleaned source files prior to integration, the spatial and metadata lookup tables, the dataset-wide statistics used in this paper, and a README that summarises the contents and serves as the primary entry point for new users.

### 4.1. Repository Structure

The released artefact is organised around two complementary forms of the dataset. The two top-level data directories (*merged* and *segregated*) correspond to these two forms as follows: the *merged* directory contains the integrated per-station tables described in Section 3.3, ready to be consumed directly by downstream applications; the *segregated* directory contains the cleaned per-source tables prior to integration, so that users wishing to apply different temporal alignment or spatial association choices can build their own integration from the same starting material. The complete directory tree of the released artefact is reproduced in Appendix A.

All data files are in plain CSV with a comma as a field separator, a period as a decimal separator, UTF-8 encoding and a header row. Missing values are encoded as empty fields. The first column of every time-series file is the temporal index, named date in the merged files and in the cleaned per-source files (the holiday calendar retains its original fecha field). The naming conventions of all station-level files are {station_id}.csv for the MITECO-anchored files and {idema}.csv or {ine_muni_code}.csv for the AEMET- and MITMA-anchored files, respectively.

### 4.2. Integrated Per-Station Files

The integrated tables in merged/hourly/ are the primary deliverable of this work. Each of the 588 files contains the full hourly time series for one MITECO air quality station over the 2022–2024 study period, joined with the lagged daily exogenous features described in Section 3.3.3. The 56 feature columns plus the date index follow the schema summarised in Table 5. The column names are stable across files, so a downstream consumer can iterate over the network without conditional logic. Pollutants not measured at a given station appear as columns of NaN; the categorical column area_type carries a station-specific constant such as *urbana de tráfico* or *rural de fondo*.

The directory merged/daily/ contains the daily aggregated variant of the integrated dataset. It is produced from the same pipeline but with the hourly air quality block collapsed to daily mean, minimum and maximum statistics prior to integration, as described in Section 3. The schema is identical to the hourly variant except that the 8 hourly pollutant columns are replaced by 24 daily columns (one mean, one minimum and one maximum per pollutant), bringing the total to 72 feature columns plus the date index at daily resolution. The variant is provided as a convenience for users for whom daily resolution is sufficient and a smaller file footprint is preferable; for any application that requires sub-daily resolution, the hourly variant is the authoritative product.

### 4.3. Segregated Per-Source Files

The directory segregated/ contains the cleaned per-source tables produced by stage 2 of the pipeline (per-source acquisition and cleaning), before any spatial pairing or temporal alignment is applied. These files reproduce the form in which each source can be obtained from its provider after the preprocessing operations described in Section 3.2. They are released alongside the merged files for users who wish to apply their own integration choices, for example, combining the air quality data with mobility at a different aggregation level, or aligning the daily blocks without the d−1 lag convention used here.

*Air quality files* are released in two resolutions. segregated/airq_hourly/{station_id} .csv contains the schema date, NO_2_, NO_*x*_, PM_10_, O_3_, CO, SO_2_, C_6_H_6_, PM_2.5_. segregated/airq_daily/{station_id}.csv contains date followed by {pollutant}_mean, {pollutant}_min and {pollutant}_max for each of the eight pollutants in the same order, for a total of 24 numeric columns; the daily aggregation follows the 75% hourly coverage rule described in Section 3.

*Meteorological files* are released in segregated/aemet/, with one CSV file per AEMET station at daily resolution and the schema date, temp_mean, precip, temp_min, temp_max, wind_speed, wind_gust, humidity_mean, humidity_max, humidity_min.

*Mobility files* are stored in three directories, with one CSV file per INE 5-digit municipality code in each. All files are at daily resolution and follow the products defined in the MITMA methodology document [36]. The schemas are segregated/people/{ine_muni}.csv with date, pop_age_0_25, pop_age_25_45, pop_age_45_65, pop_age_65_100, pop_trips_0, pop_trips_1, pop_trips_2, pop_trips_2plus, pop_mobile_ratio; segregated/overnight_stays/ {ine_muni}.csv with date, stays_total, stays_visitors, stays_residents, stays_visitor_ratio; and segregated/trips/{ine_muni}.csv with date followed by the 23 trip-related variables documented in Table 3. The MITMA municipality-level zonification covers 2687 zones [36]: all 2687 zones are present in the *viajes* block, while the *personas* and *pernoctaciones* blocks cover 2569 zones each, with the 118-zone gap explained by MITMA’s privacy-driven suppression of indicators for zones where the sample size would compromise the anonymity of the underlying mobile telephony records. These privacy-driven exclusions predominantly affect very small rural municipalities. The *viajes* files released here are obtained from the original MITMA origin–destination matrices, in which each row describes trips between a pair of zones, by aggregating to the destination zone for inbound trips and to the origin zone for outbound trips; the intra-day hourly breakdown present in the source matrices (a *franja* field with 24 values) is collapsed to the daily level, so that each row of the released file represents the totals for a single municipality on a single day.

*The public holiday calendar* is released as a single file segregated/holidays_spain.csv, covering the study period with the schema fecha, año, nombre, ambito, ccaa_codigo, ccaa_nombre, codauto, es_nacional. The ambito column distinguishes national from regional holidays, and the ccaa_codigo column carries the two-letter ISO code of the Autonomous Community when applicable. The integration step described in Section 3.3.3 reduces this calendar to the two binary indicators national_holiday and regional_holiday that appear in the merged files.

### 4.4. Supporting Files and Source Code

The lookups/ directory holds three reference tables used by the pipeline and useful for any downstream geographic operation. airq_stations.csv contains the consolidated inventory of the 588 MITECO air quality stations, with station_id, name, geographic coordinates, altitude, area type classification, dominant emission source, and the INE province and municipality codes used by the pipeline to derive the mobility and holiday linkages described in Section 3.3. aemet_stations.csv contains the consolidated inventory of the 904 AEMET meteorological stations, with the AEMET station identifier (idema), name, geographic coordinates, altitude and province. aemet_station_pairs.csv contains the output of the three-tier spatial pairing procedure described in Section 3.3.1, with one row per MITECO station; columns include MITECO station_id, the assigned AEMET idema, the Haversine distance between the two in kilometres, and the tier label of the assignment (distance, province, or fallback_absolute).

The file stats/dataset_statistics.csv contains the per-variable summary statistics used to populate Table 1, Table 2 and Table 3 of this paper. The file is in long format, with one row per (dataset, variable, metric) triple, and its supported metrics include n_total, n_missing, pct_missing, mean, std., min, q25, median, q75 and max. The file is provided so that users can reproduce the per-variable summaries reported in this paper and verify any custom statistics computed on the released files against an external reference.

The Python source code that implements the full pipeline (per-source acquisition, cleaning, spatial pairing, temporal alignment and per-station integration) is released as an open repository on GitHub [18]. The repository tag matching the release version of the dataset is also archived on Zenodo and cross-referenced from the data record. The code is provided to allow users to regenerate the dataset, extend it in time as new monthly batches of source data become available, or adapt it to other countries with comparable open data infrastructure.

## 5. Technical Validation

This section documents the technical checks applied to verify that the integrated dataset behaves as expected given the characteristics of the four source systems. The validation is organised in three threads: the spatial quality of the MITECO–AEMET station pairing, the internal consistency of each source over time and across stations, and the coherence of the cross-source signal.

### 5.1. Spatial Pairing Quality

**Figure 8:**
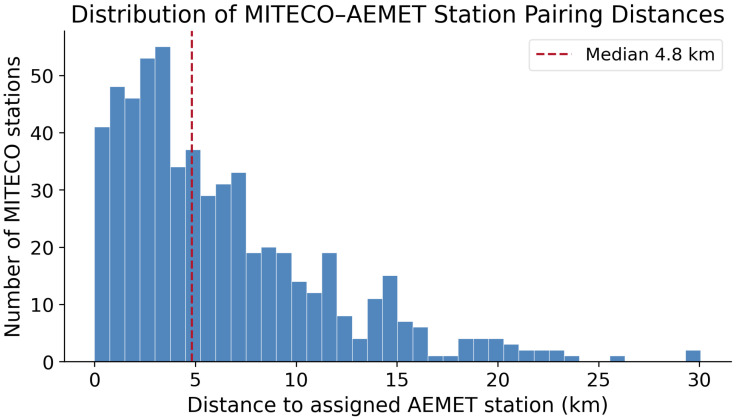
Distribution of Haversine distances (km) between the paired MITECO air quality stations and their assigned AEMET meteorological stations across the 588 pairs in the network. The dashed line marks the median distance of 4.8 km.

The three-tier pairing rule described in Section 3.3.1 assigns to each MITECO station the nearest available AEMET meteorological station. A key question for the usability of the meteorological block is how close the assigned pairs actually are in practice. Figure 8 shows the empirical distribution of Haversine distances across the 588 pairs.

All 588 MITECO stations were matched under the first-priority tier (nearest within 50 km), so the two fallback tiers were not required for any station in the network. The median pairing distance is 4.8 km and the mean is 6.3 km, reflecting the density of the Spanish AEMET network relative to the MITECO monitoring sites. The 90th percentile lies at 14.0 km and the 99th percentile at 22.2 km; the single most distant pair in the dataset is 30.1 km apart. No pair exceeds the 50 km threshold. These distances are consistent with the mesoscale meteorological homogeneity expected within typical urban and suburban airshed zones, where temperature, humidity and wind conditions a few kilometres away can reasonably approximate those at the monitoring site. Users applying the dataset to analyses that require very precise local meteorology should consult the pairing table (released in lookups/aemet_station_pairs.csv) and may wish to exclude pairs beyond a stricter distance threshold for their specific use case.

### 5.2. Internal Consistency of Source Data

**Figure 9:**
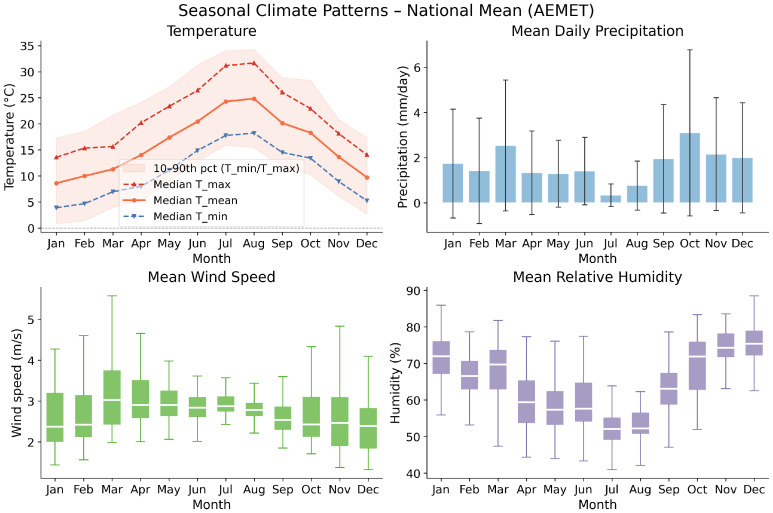
Monthly distributions of four AEMET variables across the network: median daily mean, min and max temperature with the 10th–90th percentile band (**top-left**); mean daily precipitation with error bars showing the spread across stations (**top-right**); mean wind speed as monthly box plots (**bottom-left**); and mean relative humidity as monthly box plots (**bottom-right**). Each panel pools all station-day records of the corresponding month over 2022–2024.

Figure 9 shows the monthly distribution of four AEMET variables (mean, min and max temperature; daily precipitation; mean wind speed; and mean relative humidity) across the network over the 2022–2024 study period. The patterns are consistent with the climatic characteristics of the Iberian Peninsula: median temperatures follow a smooth sinusoidal annual cycle from approximately 9 °C in January to 25 °C in July–August; mean daily precipitation is highest in October (around 3.1 mm/day with a long upper tail of comparable magnitude across stations) and lowest in July (around 0.3 mm/day), with a secondary autumn-onset rise in September and a sustained wet period from late autumn to early spring; humidity is in a clear anti-phase with temperature, peaking in late autumn and winter at around 75% and falling to around 52% in mid-summer; and wind speed varies more weakly across the year, with a slight maximum in March around 3.0 m/s. No anomalous distributional patterns that would indicate a systematic acquisition or processing error are visible in any of the variables.

**Figure 10:**
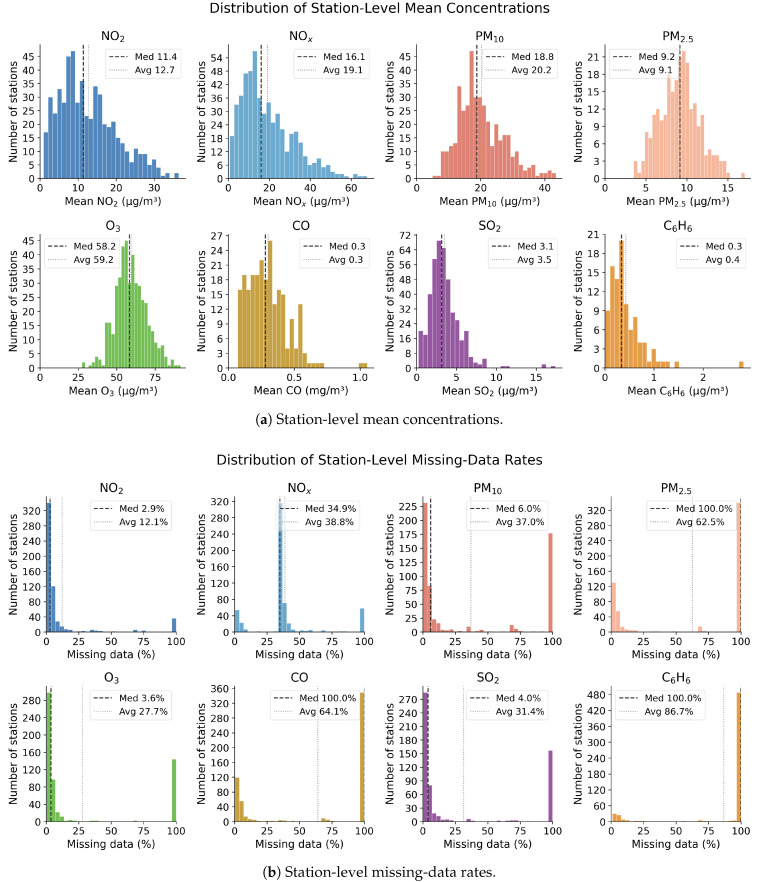
Per-station characterisation of the eight regulated pollutants across the 588 MITECO stations. (**a**) Distribution of station-level mean concentrations; the dashed and dotted vertical lines mark the across-station median and mean, respectively. (**b**) Distribution of station-level missing-data rates; the peak at 100% in each panel groups the stations that do not measure the pollutant at all, while the lower cluster groups the stations that do measure it, with their typical share of NaN records over the study period (the dashed and dotted vertical lines again mark the across-station median and mean). CO is in mg/m^3^; all other pollutants are in μg/m^3^.

Figure 10a characterises the per-station mean concentration across the network for the eight pollutants. All eight distributions are right-skewed, reflecting the typology mix of the network: rural-background and remote suburban locations populate the lower end, while urban-traffic stations in large cities populate the upper tail. The per-station median is 11.4 μg/m^3^ for NO_2_ and 16.1 μg/m^3^ for NO_*x*_, with NO_*x*_ exhibiting a noticeably longer upper tail. Particulate matter has medians of 18.8 μg/m^3^ for PM_10_ and 9.2 μg/m^3^ for PM_2.5_, both close to their respective WHO guidelines and broadly compliant with the new EU 2030 annual limits at the median station. Ozone has the most symmetric station-level distribution (median 58.2 μg/m^3^, very close to the WHO peak-season threshold of 60 μg/m^3^), consistent with its secondary photochemical origin, which homogenises concentrations across station typologies more strongly than primary-emission pollutants do. CO, SO_2_ and benzene have low absolute concentrations (medians of 0.3 mg/m^3^, 3.1 μg/m^3^ and 0.3 μg/m^3^, respectively), with right-skewed tails attributable to a handful of industrially exposed stations.

The companion distribution of station-level missing-data rates (Figure 10b) makes the coverage tiering of the network visually explicit. NO_2_ is the only pollutant that combines a low typical missingness (median around 3% among the stations that report it) with a small mass at 100% (only 36 of the 588 stations do not measure it). O_3_ and SO_2_ show similarly low typical missingness on the lower cluster (medians of 2.7% and 2.6%, respectively), but the mass at 100% is substantial in both cases: about 24% of the network does not measure O_3_ and about 27% does not measure SO_2_. NO_*x*_ has a higher typical missingness, with the bulk of the distribution clustered around 35% rather than near zero; this reflects the partial-period coverage already visible in Figure 2, where the network-wide NO_*x*_ series only stabilises towards the end of 2022, indicating that many stations began reporting this pollutant part-way through the study period. PM_10_ shows a central peak around 3% missingness on the lower cluster, with an upper quartile near 7%, together with a large secondary mass at 100% (177 stations, about 30% of the network, do not measure it). The remaining three pollutants (PM_2.5_, CO and C_6_H_6_) display the most pronounced bimodality, with a station-level median of 100% and a heavy concentration of stations at the upper end: 58%, 59% and 83% of the network respectively report no data at all for these pollutants. Users intending to work with PM_2.5_, CO or benzene should therefore expect to encounter a substantial number of stations (more than half of those in the network) with no measurement available for the pollutant of interest, regardless of how the data are subsequently processed.

### 5.3. Cross-Source Consistency

**Figure 11:**
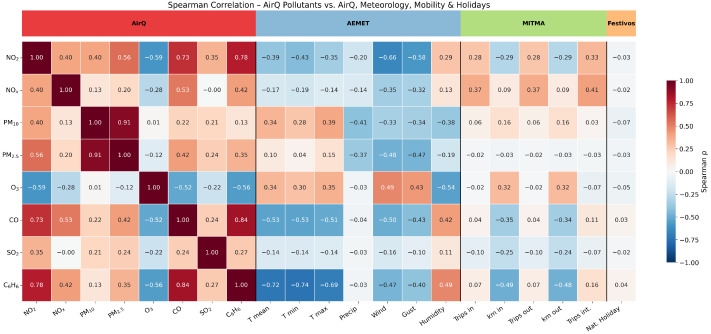
Spearman rank correlation matrix between the eight air quality pollutants (rows) and the integrated set of predictors (columns), grouped by source block: AirQ (pollutant-to-pollutant relationships), AEMET (meteorology), MITMA (mobility), and Holidays (national-holiday indicator). Correlations are computed at daily resolution across all station-day records of the network over 2022–2024.

Figure 11 shows the Spearman rank correlation matrix between the eight air quality pollutants and the full set of predictors of the integrated dataset: the air quality block itself (for the pollutant-to-pollutant relationships), the AEMET meteorological block, the MITMA mobility block at the municipal level, and the holiday indicators. Correlations are computed at daily resolution across all station-day records of the network. A rank-based measure is preferred over a linear (Pearson) coefficient because several variables (precipitation, trip counts, PM_10_) are heavily right-skewed and contain large episodic outliers that would otherwise dominate the linear coefficient.

Because the matrix is computed over more than half a million station-day records, the sampling uncertainty of each coefficient is very small and essentially every entry differs from zero at conventional significance levels (p<0.001). Statistical significance is therefore not a discriminating criterion in this figure, and the discussion below focuses instead on the sign and the effect size of each association. The differences that matter are between blocks rather than between individual cells: strong within-block coupling among the combustion pollutants and among the particulate fractions, a coherent meteorological signature that reverses sign between the combustion species and ozone, and markedly weaker mobility and holiday couplings.

The sign and approximate magnitude of every meaningful correlation are consistent with established physical and behavioural mechanisms. Within the air quality block, the strongest associations are the structural couplings between particulate matter fractions (PM_10_–PM_2.5_, ρ=0.91) and between the combustion-related pollutants, which share emission sources and meteorological controls (NO_2_–CO at ρ=0.73, NO_2_–C_6_H_6_ at ρ=0.78, CO–C_6_H_6_ at ρ=0.84). Ozone is negatively correlated with all the combustion-related pollutants (ρ between −0.28 and −0.59), as expected from the titration of freshly emitted NO and from the inverse seasonality of photochemical production and combustion emissions; its association with the particulate fractions is much weaker (close to zero for PM_10_ and around −0.12 for PM_2.5_), reflecting the fact that mineral dust and resuspended particles do not participate in the photochemical balance that drives the NO–O_3_ relationship.

The cross-correlations with the meteorological block reproduce the physically expected patterns. NO_2_ is strongly negatively correlated with wind speed (ρ=−0.66) and wind gust (ρ=−0.58), reflecting the ventilation effect that disperses locally emitted pollutants. Benzene shows the same dispersion signature (ρ=−0.47 with wind), reinforced by a strong negative correlation with temperature (ρ between −0.69 and −0.74) that captures the winter accumulation of combustion pollutants under cold-stagnation regimes. Ozone shows the inverse meteorological signature: positively correlated with temperature and wind, and negatively correlated with humidity (ρ=−0.54). PM_10_ correlates positively with daily maximum temperature (ρ=0.39) and negatively with precipitation (ρ=−0.41), consistent with soil resuspension during dry warm conditions and with wet deposition of coarse particles.

The mobility block contributes weaker correlations whose sign depends on which family of mobility indicators is considered. Trip counts (trips_inbound, trips_outbound and trips_internal) correlate positively with the locally emitted combustion pollutants NO_2_ and NO_*x*_ (ρ between 0.28 and 0.41), in line with their direct origin in road traffic. Trip kilometres (trips_inbound_km and trips_outbound_km), by contrast, correlate negatively with CO and C_6_H_6_ (ρ between −0.34 and −0.49): municipalities with longer typical incoming or outgoing trips are predominantly the less densely populated ones, which have systematically lower baseline concentrations of these combustion pollutants regardless of their daily mobility load. The same spatial gradient also produces a positive correlation of similar magnitude between ozone and trip kilometres (ρ≈0.32), since rural municipalities accumulate less NO and therefore titrate less of the available ozone. These correlations are further diluted by the daily mean aggregation and by the fact that all stations within a municipality share the same mobility record. The national-holiday indicator yields only marginal coefficients in the matrix-level view.

The absence of anomalous correlations in the matrix, in particular the lack of any sign inversion against expectation for the meteorological and pollutant-to-pollutant blocks, provides confidence that the spatial pairing procedure and the daily-to-hourly expansion have not introduced systematic distortions in the link between the four sources.

**Figure 12:**
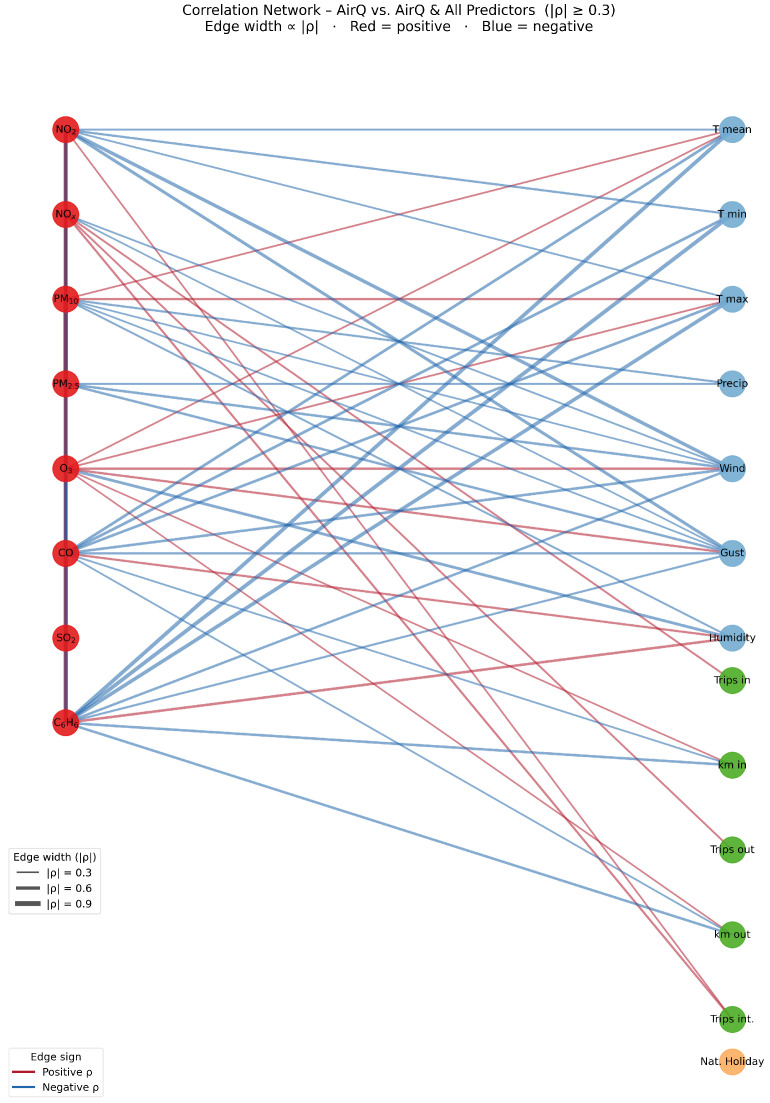
Bipartite correlation network between the eight air quality pollutants (**left**) and the AEMET, MITMA and holiday predictors (**right**). An edge is drawn whenever the absolute Spearman rank correlation exceeds 0.3; edge width is proportional to |ρ| and edge colour indicates the sign of the correlation (red: positive; blue: negative). The edge width legend reports the line thickness corresponding to |ρ|=0.3, 0.6 and 0.9.

For readability of the strongest signals, Figure 12 represents the same matrix as a bipartite network in which the eight pollutants are connected to the meteorological, mobility and holiday predictors whenever the absolute Spearman coefficient exceeds 0.3. Edge width is proportional to |ρ| and edge colour distinguishes positive (red) from negative (blue) correlations. The thickness of each edge therefore encodes the strength of the association on a linear scale: a thicker line denotes a larger absolute rank correlation, and the reference widths corresponding to |ρ|=0.3, 0.6 and 0.9 are shown in the figure legend so that the visual prominence of an edge can be read back directly to the coefficient reported in the heatmap of Figure 11. Edges below |ρ|=0.3 are omitted to keep only the associations that are both statistically robust and of practically relevant magnitude. The network view exposes three groups of nodes whose behaviour is qualitatively different. The combustion pollutants (NO_2_, NO_*x*_, CO, C_6_H_6_) share a dense bundle of negative edges to wind speed, wind gust and temperature, in line with their dispersion-driven nature, but split apart in their mobility coupling: NO_2_ and NO_*x*_ link positively to trip counts (internal, inbound and outbound), whereas CO and C_6_H_6_ link negatively to trip kilometres, for the reasons discussed in the heatmap analysis above. Ozone shows the meteorological mirror image (positive edges to wind speed, wind gust and temperature; a strong negative edge to humidity; and negative edges to the three combustion pollutants that pass the 0.3 threshold), reflecting its photochemical secondary origin; it also exhibits positive edges to incoming and outgoing trip kilometres of similar magnitude to the CO and C_6_H_6_ ones but of opposite sign, consistent with the urban-to-rural spatial gradient already identified. PM_10_, PM_2.5_ and SO_2_ are more selectively integrated into the network: PM_10_ connects to precipitation, temperature, humidity and wind speed at modest strength; PM_2.5_ is tightly coupled to PM_10_ (ρ=0.91) but also shows substantial edges to NO_2_, CO, wind speed and wind gust; and SO_2_ has no edge above the 0.3 threshold to any external predictor, consistent with its dominant industrial-source profile. The national-holiday indicator shows no significant correlations above the 0.3 threshold; a finding consistent with its diffuse rank-correlation signature across stations, as well as with the visible day-type effect documented below, where the matrix-level coefficient is too diffuse to surface as a network edge.

We stress that the relationships summarised in Figure 11 and Figure 12 are associational and are not intended to support causal inference: the network encodes rank co-variation between contemporaneous daily series, not directed chemical or behavioural pathways, and shared seasonality or common spatial gradients can induce correlations between variables that are not mechanistically linked. The signs and relative magnitudes are reported here only as a cross-source consistency check. Each interpretation offered above (the titration of ozone by freshly emitted NO, the dispersion of locally emitted pollutants by wind, the winter accumulation of combustion species under cold-stagnation regimes, and the weekend ozone effect) coincides with mechanisms that are already well established in the atmospheric chemistry and air quality literature [6,10,12,13], and is presented as evidence that the integrated dataset reproduces known physics rather than as a novel causal claim derived from the correlation structure itself.

**Figure 13:**
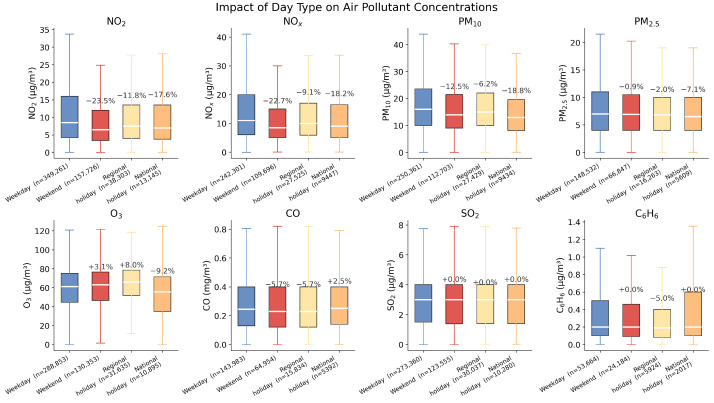
Distribution of hourly concentrations of the eight regulated pollutants stratified by day type: working weekday, weekend, regional public holiday (a holiday in at least one Autonomous Community but not nationwide), and national public holiday. The percentage above each non-weekday box reports the change in median concentration relative to the weekday baseline. Box plots summarise all station-hour records of each type over 2022–2024. CO is in mg/m^3^; all other pollutants are in μg/m^3^.

Figure 13 shows the distribution of the eight pollutants stratified by day type: working weekday, weekend, regional public holiday (a holiday in at least one Autonomous Community but not nationwide) and national public holiday. The percentage above each non-weekday box reports the change in median concentration relative to the weekday baseline.

The combustion-related pollutants display the clearest signal. For NO_2_, the weekend reduction is the largest (−23.5%), followed by the national-holiday reduction (−17.6%) and the regional-holiday reduction (−11.8%); NO_*x*_ follows the same ordering (−22.7%, −18.2% and −9.1%). The signal is consistent with the dominant role of commuting and commercial road traffic in NO_*x*_ emissions, which weakens visibly on non-working days. Particulate matter responds more weakly to day type (PM_10_: −12.5% on weekends, −18.8% on national holidays; PM_2.5_: −0.9% on weekends, −7.1% on national holidays), reflecting its broader and less commuter-driven source mix. Carbon monoxide shows a small weekend reduction (−5.7%), and benzene a small regional-holiday reduction (−5.0%), consistent with their lower absolute concentrations and noisier station coverage. SO_2_ is essentially flat across day types, in line with its industrial-source dominance and detection-limit-bounded distribution.

Ozone moves in the opposite direction (+3.1% on weekends, +8.0% on regional holidays, −9.2% on national holidays). The positive weekend and regional-holiday signal shows the well-documented weekend ozone effect: the reduction in fresh NO emissions weakens the titration of ambient ozone and allows higher peak concentrations to persist. The negative national-holiday signal most likely reflects the seasonal concentration of national holidays around late autumn and Christmas, when low temperatures and weak photochemistry depress baseline ozone irrespective of the working-day calendar.

The signal is interpretable on two levels. First, the magnitude and direction of every day-type effect are consistent with the dominant role of road traffic in primary urban pollution and with the chemistry of secondary ozone formation. Second, and more importantly for the dataset itself, the distinct reduction observed on regional holidays (days that are public holidays in the Autonomous Community of the station but not nationwide) demonstrates that the province-to-CCAA mapping described in Section 3.3 correctly assigns the relevant regional calendar to each station; had the regional flag been miscoded or assigned to the wrong stations, the regional-holiday box would be statistically indistinguishable from the weekday box. This subsection therefore validates both the calendar-source integration and the spatial association of holidays to stations.

A final cross-source consistency check is provided by the most prominent episodic feature of the air quality record. The PM_10_ spike on 15 March 2022 visible in Figure 2 reaches a network-wide daily median of 54 μg/m^3^, more than three times the typical baseline of around 15 μg/m^3^, and coincides with a well-documented Saharan dust intrusion over the Iberian Peninsula that generated air quality alerts across multiple Autonomous Communities. PM_2.5_ shows a smaller but coincident peak on the same dates (reaching 16 μg/m^3^ against a baseline near 9 μg/m^3^), consistent with the fine-particle fraction of the transported dust load. The appearance of the event as the single most pronounced network-wide particulate exceedance in the three-year record, affecting stations across all area types and both the Mediterranean and Atlantic sides of the Peninsula, is consistent with the origin and spatial extent of the event as reported at the time. None of the gaseous pollutants in the dataset (NO_2_, NO_*x*_, O_3_, SO_2_, CO, C_6_H_6_) show a corresponding spike on the same dates, as expected given that mineral dust transport has no direct relationship to local combustion or to photochemical production; this confirms that the eight pollutant series in the dataset are not cross-contaminated or otherwise spuriously coupled, and that the integrated time axis preserves the event in the pollutants where it is physically expected.

## 6. Usage Notes

This section provides practical guidance for users of the dataset. It covers loading and typical applications, recommended preprocessing for common downstream tasks, known characteristics that may affect specific analyses, and a brief account of the preliminary modelling experiments that informed the design of the release.

### 6.1. Loading and Suggested Applications

The dataset is distributed as plain CSV files that can be loaded with standard data analysis libraries. A single station in the merged hourly variant can be read with pandas in a single call, indexing the table by the date column and selecting the station by its MITECO code (listed in lookups/airq_stations.csv). Users wishing to assemble a panel across the network can iterate over the file list of the corresponding directory.

The combination of sources released here supports several research lines that would require substantial duplicated acquisition work to reproduce from the four providers independently. The most direct application is operational air quality forecasting at the station level under a multi-source feature set. Network-level comparative analyses are also natural: for example, the systematic comparison of pollutant levels across area types (urban traffic, urban background, suburban, rural background) or across Autonomous Communities, and the climatological characterisation of pollutant concentrations under different meteorological regimes. The mobility block is particularly suited to the study of activity-related drivers of urban air pollution at the municipality scale, including weekend and holiday effects, as well as the joint analysis of mobility patterns with environmental health outcomes. The dataset also provides a substrate for cross-validation of dispersion or chemistry-transport models against a dense network of in situ measurements over three full years.

### 6.2. Recommended Filtering and Preprocessing

Not all stations are equally usable for every purpose. For forecasting applications that train per-station models, we recommend filtering out stations with insufficient coverage on the target pollutant before splitting the data into training and evaluation periods. A coverage threshold of approximately 13,000 valid hours over the three-year period (around 50% of the total) is a reasonable default for NO_2_ and PM_10_; stricter thresholds may be appropriate for shorter training windows or for the less widely reported pollutants.

The dataset is explicitly intended to support machine learning applications, and the missing-data patterns documented in Section 5 have direct implications for that use; we therefore make the following guidance explicit. The coverage threshold recommended above for station selection (roughly 13,000 valid hours, about half the study period) should be evaluated separately for each target pollutant because coverage varies sharply across the eight species (Table 1, Figure 10b). Below that level, two complementary strategies are advisable. For the short, sporadic gaps that characterise the well-covered pollutants (NO_2_, NO_*x*_, O_3_ and PM_10_), whose median per-station missingness is below 4%, simple time-aware imputation, such as linear or spline interpolation over short windows, together with masking of the imputed positions so that the training loss is computed only on observed values, is generally sufficient and is preferable to discarding whole sequences. For the sparsely reported pollutants (PM_2.5_, CO and C_6_H_6_), where the dominant pattern is entire stations with no measurement rather than scattered gaps, cross-station imputation is not advisable and we instead recommend restricting analysis to the subset of stations that actually report the target. We deliberately release the data without imputation so that users retain full control over these choices, and we recommend reporting per-station coverage alongside any model results so that predictive performance is not confounded with data availability.

Users for whom hourly resolution is not required may prefer the daily aggregated variant in merged/daily/, which has approximately one twenty-fourth of the file size of the hourly variant. It replaces the eight hourly pollutant columns with their daily mean, minimum and maximum aggregates (24 columns in total) and keeps every other block of the feature schema unchanged. The daily variant offers two further advantages for users whose downstream model already operates at daily resolution. First, it does not contain the stepwise pattern that the daily-to-hourly expansion of the exogenous features introduces in the hourly tables, since every feature block in the daily variant is sampled at the same temporal resolution. Second, it is built without the one-day lag of the meteorological and mobility blocks, so those records refer to the same closed calendar day as the air quality aggregates and no information is discarded for the sake of operational realism.

The one-day lag (d−1) applied to the meteorological and mobility blocks in the hourly variant reflects the operational forecasting convention where exogenous information from day *d* only becomes available the following day. Users with access to nowcast data, or who wish to study contemporaneous rather than predictive relationships between mobility and air quality at hourly resolution, can recover the unlagged daily values from the segregated source files in segregated/ and re-join them without the temporal shift. The daily variant of the integrated dataset already provides the unlagged join for users whose downstream analysis does not require hourly granularity.

A small number of NO_2_, NO_*x*_ and PM_2.5_ records carry slightly negative values, typically within 1 to 2 μg/m^3^ of zero, which correspond to analyser calibration drift near the detection limit. These values are released as-is to preserve the raw physical readings. Users computing distributional statistics or feeding the data to scale-sensitive models may wish to apply a positivity filter, either clipping the affected values to zero or discarding them; we did not impose either choice at the dataset level.

### 6.3. Caveats and Known Limitations

Several characteristics of the released dataset deserve explicit mention. The first concerns the spatial granularity of mobility. The MITMA indicators are released at the municipality level, so all air quality stations within the same INE municipality share identical mobility features. In large urban municipalities such as Madrid or Barcelona, this approximation hides the intra-municipal heterogeneity of mobility patterns and should be borne in mind when interpreting station-level mobility–air quality relationships within a single large city.

The second concerns the coverage profile of the air quality pollutants. NO_2_ and NO_*x*_ are reported at almost every station, but the remaining six pollutants are reported by progressively smaller subsets of the network, with C_6_H_6_ available at only a small fraction of stations (Table 1). Users should verify coverage on the specific pollutant of interest before scoping a study.

The third concerns the meaning of the trips_inbound_age_na column. This is not an additional age band but a fallback category that aggregates trips for which the traveller’s age could not be inferred from the underlying mobile telephony records. Its distribution is qualitatively different from the four genuine age-band columns (a much smaller variance and no extreme outliers), and it should not be treated as comparable.

The fourth concerns the granularity of the holiday calendar. The regional_holiday flag is assigned at the Autonomous Community level. Local festivities specific to a province, municipality or smaller administrative unit (which can have measurable effects on local activity and on air quality) are not captured by the released binary indicators.

Finally, the AEMET source records contain a small number of physically impossible values, such as a relative humidity above 100% or a daily maximum temperature of −50 °C, which are present in the published data as released by AEMET. These have been preserved unchanged in the dataset. Users running anomaly-detection or scale-sensitive analyses may wish to filter them out before processing.

### 6.4. Preliminary Modelling Experiments

In the course of developing this dataset, we ran preliminary forecasting experiments using a per-station bidirectional LSTM trained on the air quality block alone, compared against the same model trained on the full enriched feature schema (air quality, meteorology, mobility and holidays). Across the target pollutants and forecast horizons we tested, the enriched feature set did not yield a robust improvement over the air quality-only baseline. These results are not evidence against the value of the enriched feature set. They suggest that capturing the predictive content of the multi-source signal may require a model architecture that exploits the network structure across stations, for instance, a graph neural network over the spatially associated stations, or a temporal resolution different from the hourly one we tested. The daily aggregated variant of the dataset, in which the lag and the daily-to-hourly expansion of the exogenous features are both absent, is plausibly more directly amenable to this kind of experiment than the hourly variant. We release this dataset in the hope that this kind of experiment becomes more practical now that the integration work is no longer a prerequisite for it.

## 7. Conclusions

This paper describes the construction and release of an open dataset that integrates the following four sensor-derived sources covering the whole of Spain from 2022 to 2024: hourly air quality observations from the 588 stations of the MITECO network, daily meteorological summaries from AEMET, daily municipality-level mobility indicators derived from anonymised mobile telephony records published by MITMA, and a calendar of national and Autonomous Community public holidays. A pipeline reconciles the sources by pairing each MITECO station with its meteorological counterpart, attaching mobility records through the INE municipality code, and aligning the daily exogenous features to the air quality time axis (a lag-and-expand procedure for the hourly variant and a same-calendar-day join for the daily variant). The released dataset comprises approximately 15 million hourly records across 588 station files (56 columns), along with a daily resolution variant (72 columns), and is published on Zenodo under a CC BY 4.0 licence [17]; the pipeline source code is released on GitHub [18].

The contribution is intentionally a data contribution. Most published work on multi-source air quality forecasting rebuilds the integration from scratch within a single project’s pipeline and rarely releases the integrated tables; by documenting and publishing ours, we aim to lower the cost of entry for research on multi-source air quality problems at the nationwide scale, from forecasting and environmental epidemiology to the validation of dispersion and chemistry-transport models.

The technical validation reported in Section 5 indicates that the dataset behaves as expected: the 588 MITECO stations are paired with AEMET stations at a median distance of 4.8 km, the seasonal climatology of the meteorological block matches that of the Iberian Peninsula, and the cross-source Spearman correlations agree in sign and magnitude with established mechanisms. The day-type analysis recovers the expected weekday, weekend and holiday contrasts for the traffic-driven pollutants (NO_2_, NO_*x*_ and PM_10_), with the opposite-sign weekend effect for ozone, and the mid-March 2022 Saharan dust intrusion appears only in the particulate (PM_10_, PM_2.5_) series, as physically expected.

The dataset’s limitations are documented in Section 6; the most consequential are the municipality-level granularity of the mobility block, which hides intra-municipal heterogeneity, and the coverage tiering of the air quality pollutants, which leaves several regulated species available at only a fraction of the network. Future versions deposited on Zenodo will extend the temporal coverage as new MITMA batches are published and may incorporate finer-grained zonifications, and the released source code is intended to support adaptation to other countries with comparable open data infrastructure.

## Figures and Tables

**Table 1:** Per-variable summary statistics for the eight pollutants of the MITECO block, ordered by station coverage (most widely reported first). *N* is the number of station-hour records reported by stations that measure the variable, out of a theoretical maximum of 15,466,752 (588 stations × 26,304 h); *missing* is the share of those records that are NaN. Concentrations are in μg/m^3^, except for CO, which is in mg/m^3^.

Variable	Unit	N	Missing	Mean	Std.	Median	Min	Max
NO_2_	μg/m^3^	14,087,160	6.4%	12.84	14.29	8.0	−1.0	403
NO_*x*_	μg/m^3^	13,886,592	33.9%	18.83	29.68	10.5	−2.0	2785
O_3_	μg/m^3^	11,364,600	4.3%	59.21	27.86	59.9	0.0	339
SO_2_	μg/m^3^	11,016,048	6.4%	3.44	4.46	3.0	0.0	583
PM_10_	μg/m^3^	10,433,952	9.9%	20.30	28.27	15.0	0.0	3607
PM_2.5_	μg/m^3^	6,318,768	11.7%	9.02	9.80	7.0	−0.3	1024
CO	mg/m^3^	6,148,536	11.8%	0.31	0.23	0.23	0.0	13.1
C_6_H_6_	μg/m^3^	2,561,712	21.9%	0.46	1.01	0.2	0.0	200.9

**Table 2:** Per-variable summary statistics for the nine retained AEMET variables. All variables share a denominator of approximately one million station-day records over the 1096-day window; the exact *N* varies slightly between variables because not every station reports every channel for every day. The wind variables (wind_speed, wind_gust) are reported at a smaller subset of stations than the temperature, precipitation and humidity variables.

Variable	Unit	Missing	Mean	Std.	Median	Min	Max
temp_mean	°C	4.8%	16.11	7.06	15.8	−15.0	40.4
temp_min	°C	4.8%	10.60	6.86	10.4	−18.7	50.0
temp_max	°C	4.8%	21.63	8.03	21.3	−50.0	46.8
precip	mm	4.7%	1.66	6.16	0.0	0.0	710.8
wind_speed	m/s	5.1%	2.79	1.88	2.5	0.0	33.1
wind_gust	m/s	5.3%	9.64	4.08	8.9	0.0	65.6
humidity_mean	%	5.6%	64.25	18.38	64.0	1.0	100.0
humidity_min	%	5.2%	46.36	19.48	43.0	0.0	100.0
humidity_max	%	5.2%	86.45	13.96	91.0	1.0	111.0

**Table 3:** Per-variable summary statistics for the 36 MITMA variables released across the three indicator blocks. The *pernoctaciones* and *personas* blocks are computed across 2,781,949 zone-day records; the *viajes* block is computed across 2,824,332 zone-day records. Missingness is 0% for every variable. The distributions are strongly right-skewed; medians are therefore a more representative summary of the typical zone-day than means. Values above $10^{6}$ are reported with the M suffix.

Variable	Unit	Mean	Std.	Median	Max
Pernoctaciones (overnight presence)					
stays_total	persons	18,534	79,878	6201	3.4 M
stays_residents	persons	6553	8776	4625	128,276
stays_visitors	persons	11,981	80,244	640	3.4 M
stays_visitor_ratio	ratio (0–1)	0.258	0.341	0.096	1.000
Personas					(daytime presence)
pop_age_0_25	persons	4478	18,279	1458	763,209
pop_age_25_45	persons	4804	22,554	1518	961,954
pop_age_45_65	persons	5563	23,049	1908	980,847
pop_age_65_100	persons	3681	16,171	1461	696,233
pop_trips_0	persons	5078	18,619	2521	1.2 M
pop_trips_1	persons	819	3482	252	371,659
pop_trips_2	persons	3803	16,367	1309	759,272
pop_trips_2plus	persons	8827	42,286	2425	2.0 M
pop_mobile_ratio	ratio (0–1)	0.640	0.138	0.684	1.000
Viajes–aggregate trip counts and kilometres					
trips_inbound	trips	48,507	238,943	12,590	11.5 M
trips_outbound	trips	48,507	238,933	12,588	11.5 M
trips_internal	trips	27,728	194,811	2358	9.7 M
trips_inbound_km	km	463,566	1,971,094	188,015	163.4 M
trips_outbound_km	km	463,566	2,004,148	188,051	154.4 M
Inbound trips by destination activity					
trips_inbound_act_home	trips	18,345	83,060	5152	3.9 M
trips_inbound_act_frequent	trips	17,606	88,784	3971	5.0 M
trips_inbound_act_infrequent	trips	7230	43,030	1855	2.9 M
trips_inbound_act_work_study	trips	5325	27,225	1371	1.6 M
Inbound trips by household income					
trips_inbound_income_high	trips	9308	111,662	444	6.1 M
trips_inbound_income_mid	trips	30,918	130,580	8002	5.3 M
trips_inbound_income_low	trips	8281	31,438	544	1.9 M
Inbound trips by traveller age band					
trips_inbound_age_0_25	trips	8924	52,224	1026	2.5 M
trips_inbound_age_25_45	trips	10,388	69,358	989	3.4 M
trips_inbound_age_45_65	trips	11,886	72,336	1245	3.6 M
trips_inbound_age_65_100	trips	5500	35,834	575	1.8 M
trips_inbound_age_na	trips	11,810	17,061	7316	339,867
Inbound trips by traveller residency					
trips_inbound_residents	trips	46,023	230,571	11,480	11.1 M
trips_inbound_nonresidents	trips	2484	9263	853	691,003
Outbound trips by origin activity					
trips_outbound_act_home	trips	17,961	81,724	5089	4.0 M
trips_outbound_act_frequent	trips	18,093	90,741	4100	5.1 M
trips_outbound_act_infrequent	trips	7173	42,759	1799	2.9 M
trips_outbound_act_work_study	trips	5279	26,804	1361	1.5 M

**Table 4:** Source-level summary of the four processed datasets. Counts and resolutions refer to the data as published by each provider, prior to the integration step. The missing-data column for AIRQ is the range observed across the eight pollutants; for AEMET, it is the range across the nine retained variables. The MITMA record count corresponds to the *viajes* block; the *personas* and *pernoctaciones* blocks cover 2,781,949 zone-day records each.

Dataset	Provider	Resolution	Spatial Units	Records	Missing	Download
Air quality (AIRQ)	MITECO	hourly	588 stations	14,944,488	4.3–33.9%	CSV
Meteorology (AEMET)	AEMET	daily	904 stations	990,784	4.7–5.6%	Bulk API
Mobility (MITMA)	MITMA	daily	2687 zones	2,824,332	0%	CSV
Public holidays	Gov. ES	daily	19 CCAAs	230	—	Library

**Table 5:** Feature schema of the integrated per-station table. The total of 56 columns reflects the 8 air quality variables, 9 meteorological variables, 4 overnight-stay indicators, 9 people indicators, 23 trip-related indicators, 2 holiday flags and 1 area-type categorical column. Daily resolved blocks (AEMET, MITMA) are lagged by one day (d−1) and expanded to hourly resolution; the air quality block is at its native hourly resolution; the holiday flags are assigned without lag.

Source/Block	Cols	Variables
Air quality (MITECO)	8	NO_2_, NO_*x*_, O_3_, SO_2_, CO, PM_10_, PM_2.5_, C_6_H_6_. Hourly concentrations in μg/m^3^ (CO in mg/m^3^).
Meteorology (AEMET)	9	temp_mean, temp_min, temp_max, precip, wind_speed, wind_gust, humidity_mean, humidity_min, humidity_max. Daily values, lagged d−1, expanded to hourly.
Mobility, pernoctaciones (MITMA)	4	Total overnight stays, residents, visitors, visitor ratio. Daily, lagged d−1, expanded.
Mobility, personas (MITMA)	9	Population counts by age band (0–25, 25–45, 45–65, 65–100), by number of trips per day (0, 1, 2, 2+) and mobile-population ratio. Daily, lagged d−1, expanded.
Mobility, viajes (MITMA)	23	Inbound and outbound trip counts and kilometre aggregates, segmented by trip purpose, household income, traveller age band (including unknown) and residency status; plus internal trips. Daily, lagged d−1, expanded.
Holiday flags	2	Binary indicators for national and Autonomous Community holidays, assigned at time *t* without lag.
Station metadata	1	Area-type categorical label (area_type): *urbana de tráfico*, *urbana de fondo*, *suburbana*, *rural de fondo*, etc. Constant per station.

## Data Availability

The integrated multi-source dataset described in this paper is released as an open record on the Zenodo repository under a Creative Commons Attribution 4.0 International (CC BY 4.0) licence [17]. The persistent DOI of the record is 10.5281/zenodo.20196221, accessible at https://doi.org/10.5281/zenodo.20196221, and resolves to the latest published version. The Python source code of the integration pipeline is released as an open repository on GitHub [18] at https://github.com/juanbonastre/airq_enriched_dataset (accessed on 16 May 2026); the tag corresponding to the release version of the dataset is mirrored on Zenodo and cross-referenced from the data record.

## Glossary

AI	Artificial Intelligence
DL	Deep Learning
ML	Machine Learning
CNN	Convolutional Neural Network
LSTM	Long Short-Term Memory
RNN	Recurrent Neural Network
GNN	Graph Neural Network
MAE	Mean Absolute Error
RMSE	Root Mean Squared Error
MITECO	Spanish Ministry for the Ecological Transition
AEMET	Spanish State Meteorological Agency
MITMA	Spanish Ministry of Transport and Sustainable Mobility
CDR	Call Detail Record
INE	Spanish National Statistics Institute
API	Application Programming Interface
WHO	World Health Organization

## Appendix A. Repository Structure

This appendix reproduces the full directory tree of the released artefact, relocated here from the main text for readability.

The released artefact follows the directory structure shown below.
